# ETS transcription factor ELF3 (ESE‐1) is a cell cycle regulator in benign and malignant prostate

**DOI:** 10.1002/2211-5463.13417

**Published:** 2022-05-06

**Authors:** Leanne K. Archer, Fiona M. Frame, Hannah F. Walker, Alastair P. Droop, Georgina L. K. McDonald, Samuel Kucko, Daniel M. Berney, Vincent M. Mann, Matthew S. Simms, Norman J. Maitland

**Affiliations:** ^1^ 8748 Cancer Research Unit Department of Biology University of York Heslington UK; ^2^ Department of Molecular Oncology Barts Cancer Institute Queen Mary University of London UK; ^3^ 47665 Experimental Cancer Genetics Wellcome Sanger Institute Hinxton UK; ^4^ 156739 Department of Urology Castle Hill Hospital Cottingham UK

**Keywords:** cell cycle, ELF3, patient samples, prostate, prostate cancer, siRNA

## Abstract

This study aimed to elucidate the role of ELF3, an ETS family member in normal prostate growth and prostate cancer. Silencing ELF3 in both benign prostate (BPH‐1) and prostate cancer (PC3) cell lines resulted in decreased colony‐forming ability, inhibition of cell migration and reduced cell viability due to cell cycle arrest, establishing ELF3 as a cell cycle regulator. Increased ELF3 expression in more advanced prostate tumours was shown by immunostaining of tissue microarrays and from analysis of gene expression and genetic alteration studies. This study indicates that ELF3 functions not only as a part of normal prostate epithelial growth but also as a potential oncogene in advanced prostate cancers.

## Graphical abstract

In the prostate, ELF3 is expressed in committed basal (CB) cells but not stem cells (SC), transit amplifying (TA) cells or luminal (L) cells. Using siRNA to knockdown ELF3 expression results in cell cycle arrest, reduced colony formation and reduced wound healing. ELF3 is expressed in some high grade cancers and there is evidence for ELF3 amplification in metastatic cancers.

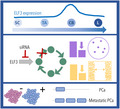

**Abbreviations**
AMACR: alpha‐methylacyl‐CoA racemase
APES: 3‐Aminopropyltriethoxysilane
AR: androgen receptor
BCA: bicinchoninic acid
BPH: benign prostatic hyperplasia
CB: committed basal
CRPC: castration‐resistant prostate cancer
DAPI: 4′,6‐diamidino‐2‐phenylindole
DAVID: database for annotation, visualization and integrated discovery
ELF3: E74‐like transcription factor‐3
ETS: E26 transformation‐specific
FCS: fetal calf serum
GO: gene ontology
LIMMA: linear models for microarray and RNA‐seq data
MDS: multidimensional scaling
NE: neuroendocrine
PCa: prostate cancer
PFA: paraformaldehyde
RIN: RNA integrity number
RIPA: radioimmunoprecipitation assay
RP: radical prostatectomy
SC: stem cell
SCM: stem cell media
TA: transit amplifying
TAC: transcriptome analysis console
TMA: tissue microarray
TRUS: transrectal ulstrasound
TURP: trans‐urethral resection of the prostate

The prototype ETS (E26 transformation‐specific) factors were originally discovered due to their integral roles in leukaemias and cancers, and of the 28 family members detected in the human genome, an increasing number of ETS family members have since been linked to various different cancers [1, 2, 3, 4, 5]. In prostate cancer (PCa), *ERG* is the most overexpressed oncogene in patient tumour samples and the *TMPRSS2‐ERG* fusion, which can be formed by translocation or interstitial deletion, is a suspected driver of tumourigenesis in about 50% of prostate cancers [6, 7, 8]. More recently, additional key roles for other members of the ETS family in PCa are emerging [9, 10, 11, 12, 13, 14].

Within the ETS transcription factor family, there is a subset of genes whose expression is limited to epithelial cells; *ESE2*, *ESE3* and *ELF3* (also known as *ESE1*) are highly related and form a distinct gene subfamily. The GGAA‐binding set of ETS factors all display important roles in epithelial cell differentiation. Notably, there is now mounting evidence that several of these factors can play a role in PCa [15, 16, 17, 18].

Two key studies addressing the role of ELF3 (E74‐like transcription factor‐3) in PCa produced conflicting results. First, ELF3 was described as a tumour repressor of PCa [12] by interfering with androgen receptor (AR) DNA binding, resulting in repression of AR target genes, which are drivers of PCa. Knocking down *ELF3* in the LNCaP cell line model led to increased cell migration and proliferation, whilst overexpression of *ELF3* in an LNCaP xenograft model repressed tumour growth. There were also some indications that ELF3 protein loss was associated with PCa progression following immunohistochemical analysis of patient tissue sections in a small patient cohort (29 patients).

In contrast, the second key study [19] described ELF3 as an oncogenic driver of PCa, based on the detection of high levels of ELF3 mRNA in patient data sets and elevated expression of ELF3 protein in 63% of 207 tumours. Only low ELF3 expression was seen in normal tissues. The proposed mechanism by which ELF3 might drive tumour progression was through a positive feedback loop with NFkappaB, which upregulates ELF3 in model systems. In addition, ELF3 overexpression in 22RV1 xenografts resulted in larger and faster growing tumours [19].

A number of proposals for the use of small molecule inhibitors of ETS factors as cancer therapies have recently been reported (reviewed by Hsing et al. [20]), but their deployment against ELF3 in PCa is critically dependent on whether the transcription factor is acting to suppress or to promote cancer cell growth. To resolve this, we examined the effects of silencing of *ELF3* in prostate epithelial cells of malignant and non‐malignant origins.

## Materials and methods

### Culture of cell lines

Cell lines were maintained at 37 °C with 5% CO_2_. BPH‐1 cells were cultured in RPMI plus 5% fetal calf serum (FCS), and PC3 cells were cultured in Hams‐F12 media plus 7% FCS. Both had 2 mm L‐Glutamine. The PC3 cell line was obtained from ATCC (CRL‐1435). The BPH‐1 cell line was gifted by Professor Simon Hayward, Director, Cancer Biology, NorthShore University HealthSystem. The PNT1a cell line was gifted from P. Berthon (currently, these cells are available from ECACC ‐ 95012614). The PNT2‐C2 cell line was obtained from ECACC (95012614). The P4E6 cells were derived in York (currently available from ECACC ‐ 10112301). The Du145 cell line was obtained from ATCC (HTB‐81). The LNCaP cell line was obtained from ATCC (CRL‐1740). The 22RV1 cell line was obtained from ATCC (CRL‐2505). The original references for all these cell lines are referenced in Fig. S2. PNT1a, PNT2‐C2, LNCaP and Du‐145 cells were cultured in RPMI plus 10% FCS.

### Culture of primary prostate epithelial cells

A detailed account of primary prostate tissue processing and cell culture is described in Frame et al. [21], including the components of stem cell media (SCM), which is used to grow primary epithelial prostate cell cultures. Informed consent (written) was obtained from all patients for tissue involved in the study. Prostate tissue was obtained from patients with ethical permission and consent by a tissue procurement officer (Ethics Number: 07/H1304/121) from Castle Hull Hospital, Cottingham, Hull. Experiments were performed in accordance with the standards set by the Declaration of Helsinki. Patient tissue was anonymised and derived from either benign prostatic hyperplasia (BPH) by transurethral resection of the prostate (TURP) or targeted needle core biopsies of tumours and adjacent normal tissue following radical prostatectomy (RP). Cancer cores were taken from palpable tumours when possible or alternatively from cancerous areas detected by MRI and transrectal ultrasound (TRUS) biopsies. Patient‐derived primary epithelial cells were cultured on collagen‐I coated 10 cm dishes (BD Biosciences, Franklin Lakes, NJ, USA) with murine STO fibroblast feeder cells (irradiated at 60 Gy) and maintained with fresh SCM every other day and passaged at a ratio of 1 : 2–1 : 4 by trypsinisation. Following the first passage after processing, STOs were no longer used. Irradiation of STOs was carried out using an X‐RAD iR225 irradiator. Notably, primary prostate epithelial cells are not luminal cells because luminal cells cannot be cultured as they are terminally differentiated; however, these primary prostate epithelial cells can be pushed to differentiate in differing culture conditions [22, 23] and several studies have shown that the primary cells from cancers are different to the primary cells from BPH, using measurements such as invasiveness, drug response and the presence of TMPRSS2:ERG fusion, thus making them a valid model to study PCa [24].

### Selection of transit amplifying (TA) and committed basal (CB) cells

Transit amplifying and CB cells were enriched from patient‐derived primary prostate epithelial cell cultures using cell surface markers, as described previously by Collins et al. and Frame et al. [21, 25]. TA cells are defined as α_2_β_1_‐integrin^high^ and CB cells are α_2_β_1_‐integrin^low^. Thus, α_2_β_1_‐integrin expression allows enrichment of TA cells by rapid collagen adherence.

### siRNA transfection of prostate cell lines

Silencer Select siRNAs (ThermoFisher Scientific, Waltham, MA, USA) were used to knock down ELF3 expression in prostate cell lines and patient‐derived primary epithelial cells. Cells were plated at a density of 4 × 10^4^ – 3 × 10^5^ to adhere overnight. A 10 μm stock of ELF3 siRNA (siELF3, Assay ID: s4623) or scrambled siRNA (siSCR, Negative control no. 1) was added to OptiMEM reduced serum medium to produce mix 1 and Lipofectamine RNAiMAX (Invitrogen, Waltham, MA, USA) was added to OptiMEM to produce mix 2. The mixtures were added together at a ratio of 1 : 1 and incubated for 15 min at room temperature. The transfection mix was then added dropwise into media of wells. Cells were harvested at various time points for protein analysis and functional assays.

### Preparation of RNA for microarray analysis

RNA was extracted from cells using the RNeasy Mini Kit (QIAGEN, Germantown, MA, USA). Sample sets were in triplicate and included un‐transfected, mock transfected, siSCR and siELF3 transfected BPH‐1 and PC3 cells. The RNA integrity number (RIN) of all samples was 9.8 or above (analysed by an Agilent Bioanalyzer, Santa Clara, CA, USA). Each sample was carried out in duplicate to also obtain protein for western blot analysis. ELF3 knockdown was verified at the protein level before microarray analysis.

### Microarray data analysis

An Affymetrix Clariom D gene expression microarray was performed by Eurofins Genomics on RNA. transcriptome analysis console (tac) Software 4.0 was used to analyse the data (ThermoFisher Scientific). A significance threshold of twofold increase or decrease and a *P*‐value < 0.05 was used. Gene ontology (GO) analyses were carried out using the Database for Annotation, Visualization and Integrated Discovery (DAVID) and visualised using REViGO. Data were also analysed using LIMMA (Linear Models for Microarray and RNA‐seq Data) within the R numerical environment, with a false discovery rate threshold of 0.025 after empirical Bayes smoothing of the standard errors [26, 27]. Multidimensional scaling (MDS) showed that BPH‐1 and PC3 cells were different from each other and samples within each set were consistent and could therefore be compared.

### Protein extraction and quantification

Cells were harvested using trypsin, and the resulting pellets were lysed in radioimmunoprecipitation assay (RIPA) buffer or CytoBuster lysis buffer (Novagen, Malvern, Worcestershire, UK) with the addition of protease inhibitors (cOmplete™, Mini, EDTA‐free Protease Inhibitor Cocktail, Roche, Munich, Germany). Phosphatase inhibitors were also added if appropriate (PhosSTOP Roche). A Bicinchoninic acid (BCA) assay (Thermofisher Scientific) was used to quantify protein concentration from whole‐cell lysates according to the manufacturer’s instructions.

### SDS‐PAGE gel electrophoresis and western blotting

8–12% Tris‐SDS acrylamide gels were prepared using the Bio‐Rad (Watford, Hertfordshire, UK) Protean II system. 20–30 µg of protein lysate was added to 4× Laemmli Sample Buffer (Bio‐Rad) and heated to 95 °C for 5 min. Samples were added to wells with the Precision Plus Protein Kaleidoscope ladder (Bio‐Rad) in a separate lane. Proteins were subjected to electrophoresis and then transferred to Immobilon‐P membrane (Millipore, Watford, Hertfordshire, UK) using the Bio‐Rad Protean II system in transfer buffer [48 mm Tris, 39 mm glycine, 10% (v/v) methanol]. Membranes were then blocked with 5% (w/v) non‐fat skimmed milk (Marvel, Marvel milk, Premier Foods, St Albans, Hertfordshire, UK) at room temperature for 1 h. Primary antibody diluted in 1% (w/v) Marvel or 5% (w/v) BSA in TBST [150 mm NaCl, 50 mm Tris‐HCl pH 7.5, 0.1% (v/v) Tween 20] was added and incubated overnight at 4 °C. The following day, membranes were washed in TBST buffer three times for 5 min. Membranes were incubated with secondary antibodies (goat anti‐rabbit IgG HRP‐linked, Cell Signalling Technologies or goat anti‐mouse IgG HRP‐linked, Affinipure, Baltimore, PA, USA) for 1 h at room temperature at a concentration of 1 : 10 000. After washing in TBST, the BM Chemiluminescence Blotting Substrate (Roche) was used to develop the membranes. Membranes were exposed to hyperfilm ECL (GE Healthcare, Chicago, IL, USA) and processed using an X‐ray processor (SRX‐101A, Konica Minolta, Chiyoda City, Tokyo, Japan). A list of antibodies used is presented in Table S1.

### Paraffin‐embedding and sectioning of tissue

Small segments of prostate tissue biopsies and TURPs were submerged in formalin overnight. The following day, the tissue was moved to a histocassette and placed in 70% ethanol until paraffin‐embedding. Prepared tissues in histocassettes were transferred from storage in 70% ethanol into fresh 70% ethanol for 10 min. Histocassettes were then placed in 100% ethanol 3 × 10 min, propan‐2‐ol 2 × 10 min and xylene 4 × 10 min. Four paraffin wax pots were melted in an oven at 60 °C, and the histocassettes were placed in each pot for 15 min consecutively. The samples were removed from the histocassette, then placed and orientated in metal moulds partially filled with molten wax. The lid of the histocassette was placed on top of the sample, and the mould was filled up with molten wax. The samples were set on a cold plate for 20 min and removed from the mould. Samples were stored at room temperature until sectioning. SuperFrost Plus Slides (ThermoFisher Scientific) were first coated in 3‐Aminopropyltriethoxysilane (APES) as follows: submerged in 2% APES in acetone for 1 min, 2× acetone washes, 2× distilled water washes and placed on a slide dryer overnight. Paraffin‐embedded tissues were sectioned on a Leica RM2235 microtome. Sections were 5 μm thick and placed on APES‐coated slides and placed on a slide dryer overnight.

### Immunohistochemistry (IHC) of paraffin‐embedded prostate tissue

Paraffin‐embedded prostate tissue sections were baked at 45 °C for 20 min on a slide dryer. Deparaffinisation and rehydration were performed by immersing slides in the following baths; xylene 2 × 10 min, xylene 1 × 1 min, 100% ethanol 3 × 1 min and 70% ethanol 1 × 1 min. Slides were then washed for 5 min under running tap water. Heat‐induced epitope retrieval (HIER) was carried out in sodium citrate buffer (pH 6) using the 2100 Antigen Retriever (Aptum Biologics, Southampton, Hampshire, USA) pressure cooker. Slides were washed three times in PBS for 5 min on an orbital shaker. A PAP pen (Dako, Santa Clara, CA, USA) was used to create a hydrophobic barrier around each tissue section, which was subsequently blocked in 10% (v/v) FCS in PBS for 1 h at room temperature in a dark moist box. The block was removed and sections were incubated in primary antibody diluted in 10% (v/v) FCS in PBS overnight at 4 °C in the box. The following day, slides were washed three times in PBS for 5 min and treated with 3% (v/v) hydrogen peroxide in PBS for 30 min to remove endogenous peroxidases. Slides were rinsed in PBS and then incubated with the secondary biotinylated antibody diluted in 10% (v/v) FCS in PBS for 30 min at room temperature. After washing three times in PBS for 5 min, they were then incubated with the tertiary antibody (streptavidin‐HRP) diluted in 10% FCS for 30 min at room temperature. Slides were washed twice in PBS for 5 min, and sections were then incubated with diaminobenzidene (ImmPACT DAB peroxidase substrate, Vector Laboratories, Burlingame, CA, USA) until sections started to turn brown (10 s – 2 min). Following rinsing in distilled water and then running tap water for 5 min, sections were counterstained with haematoxylin for 3 s, rinsed with water and then dehydrated through the following baths; 70% ethanol 1 × 1 min, 100% ethanol 3 × 1 min and xylene 2 × 1 min. The slides were then mounted with DPX (Sigma‐Aldrich) and covered with a coverslip. Primary and secondary antibodies used are shown in Table S2.

### IHC using the ImmPRESS excel amplified HRP polymer staining kit

The ImmPRESS Excel Amplified HRP Polymer Staining Kit (anti‐rabbit IgG kit: MP‐7601, anti‐mouse IgG kit: MP7602, Vector Laboratories) was employed to amplify signal of potentially weakly expressed antigens. Baking, deparaffinisation, hydration and antigen retrieval was carried out as described above. All further reagents used were provided in the kit. The following day, sections were incubated with BLOXALL blocking solution for 10 min to quench endogenous peroxide activity and subsequently washed in running water for 10 min. Sections were then blocked in 2.5% normal horse serum for 20 min. The block was removed and sections were incubated in primary antibody diluted in 2.5% normal horse serum overnight at 4 °C in a dark moist box. Slides were washed three times in TBST for 5 min and then incubated with Amplifier Antibody for 15 min followed by another two washes in TBST for 5 min. Sections were then incubated with ImmPRESS Excel Reagent for 30 min and washed once in TBST and then in dH_2_O for 5 min. Equal volumes of ImmPACT DAB *EqV* Reagent 1 and 2 were combined and added to sections until they turned brown (10 s – 2 min). Sections were then rinsed in dH_2_O followed by running tap water. Slides were counterstained with haematoxylin for 3 s, rinsed with water and dehydrated and mounted as above.

### Immunocytochemistry (ICC) – fixed cells

Cells were plated onto 8‐well chamber slides and left to adhere overnight (~ 10 000 cells per well). Following two PBS washes, cells were then fixed with either 200 μL 4% paraformaldehyde (PFA) pH 7.4 for 10 min at room temperature or 1 : 1 methanol:acetone for 30 s at room temperature. After washing in PBS three times for 5 min, cells were permeabilised using 200 μL 0.5% (v/v) Triton X‐100 in PBS for 10 min at room temperature. Following a further three PBS washes for 5 min, cells were then blocked in 10% (v/v) goat serum in PBS for 1 h at room temperature. Cells were then incubated with primary antibody diluted in 10% goat serum overnight at 4 °C. Secondary antibody only controls were performed by incubating in 10% goat serum only overnight. Primary and secondary antibodies used are shown in Table S3. The following day, slides were washed three times in PBS for 5 min and incubated with 200 μL secondary antibody at a dilution of 1 : 1000 in 10% goat serum. Cells were washed a final five times with PBS for 5 min whilst protected from light and the chambers were then removed. Nuclear staining was performed using Vectashield mounting medium with 4′,6‐diamidino‐2‐phenylindole (DAPI) (Vector Laboratories) and slides covered with a coverslip (22 × 50 mm, SLS) and sealed. Slides were analysed on a Leica DMIL LED fluorescent microscope or a Zeiss LSM 880 confocal microscope.

### Cell viability alamarBlue assay

Cells were plated in 24‐well plates in triplicate at a density of 4 × 10^4^ BPH‐1 cells per well and 6 × 10^4^ PC3 cells per well and left to adhere overnight in 500 μL media. The following day, cells were transfected to knock down ELF3. At days 1, 2 and 3, 50 μL of alamarBlue reagent (diluted 1 : 10 in the corresponding media for each cell line) was added to each well and incubated at 37 °C for 2 h. Fluorescence intensity was determined using a microplate reader (Polarstar Optima, BMG Labtech, Ortenberg, Germany) at excitation/emission values of 544/590 nm. As cells were approaching confluency at day 3, they were trypsinised and replated at the original plating density and viability was analysed on days 4, 5 and 6.

### Cell adhesion assay

To assess the effect of ELF3 knockdown on cell adhesion, BPH‐1 cells, following knockdown, were detached by incubation with trypsin and replated in a 6‐well plate at three different densities; low (40 000), medium (100 000) and high (300 000). Cells were left to adhere for 4 h at which point non‐adherent cells were washed off and adherent cells were trypsinised and counted using the Vi‐Cell Cell Viability Analyser (Beckman Coulter, Brea, CA, USA).

### Wound healing assay

Cells were plated onto 12‐well plates in triplicate (2 × 10^5^ BPH‐1, 2.5 × 10^5^ PC3 per well) and left to adhere overnight. Cells were transfected the following day to knock down ELF3. At 24 h post‐transfection, a wound was made in the confluent monolayer. Images were taken at zero hours using an EVOS XL transmitted light microscope (AMG) at 10×. The end point was determined by monitoring the wounds until the first triplicate set of one condition (mock, siSCR or siELF3) had closed. The mean width of the wounds was determined between 10 points using Image J software [28]. Per cent wound closure was calculated at the end point relative to the zero hour images.

### Colony‐forming assay

Colony‐forming assays were carried out by plating 200 BPH‐1 or PC3 cells into 12‐well plates in triplicate. Cells were plated 24 h after knockdown treatment (mock, siSCR or siELF3) and were supplemented with fresh media every 2 days. At day 7, cells were stained with crystal violet [1% (w/v) crystal violet, 10% (v/v) ethanol in PBS]. Colonies consisting of > 32 cells were counted (representative of 5 population doublings) [29, 30].

### Cell cycle analysis

Cell cycle analysis was carried out by flow cytometry using the Click‐iT Plus EdU Pacific Blue Flow Cytometry Kit (ThermoFisher Scientific) and propidium iodide according to the manufacturer’s instructions. Briefly, 4 × 10^4^ BPH‐1 and 8 × 10^4^ PC3 cells were plated onto a 12‐well plates. The following day, cells were transfected to knock down ELF3. On days 1, 2 and 3, post‐transfection cells were treated with 10 μm EdU. After 4 h, cells were harvested and washed in 3 mL 1% BSA in PBS. The pellet was resuspended in 100 μL Click‐iT fixative and incubated at room temperature for 15 min away from light. Cells were washed in 3 mL 1% BSA in PBS and then stored in PBS at 4 °C until they were to be analysed by flow cytometry. Cells were centrifuged and resuspended in 100 μL of 1× Click‐iT saponin‐based permeabilisation and wash reagent and incubated at room temperature for 15 min. 500 μL of the Click‐iT Plus reaction cocktail (437.5 μL PBS, 10 μL copper protectant, 2.5 μL fluorescent dye picolyl azide, 50 μL reaction buffer additive) was added to each tube, mixed well and incubated at room temperature for 30 min away from light. Cells were then washed in 1× Click‐iT saponin‐based permeabilisation and wash reagent, centrifuged and resuspended in 400 μL of the same reagent. A total of 50 μL of RNase A (1 mg·mL^−1^, Sigma) and 50 μL propidium iodide (400 μg·mL^−1^, Sigma) were added to each sample and incubated at 37 °C for 30 min before analysing on the flow cytometer.

Results were analysed using Summit software. The cell population of interest was gated using a FS Lin/SS Log histogram, and doublets were excluded using a PE‐Texas Red Lin/PE‐Texas‐Red Area histogram. PE‐Texas Red Area/Violet 1 Log was used to determine the proportion of cells in G1, S and G2 phases of the cell cycle. At least 10 000 events were collected for significance.

### CRISPR protocol

To permanently knock down ELF3 in the BPH‐1 cell line, CRISPR technology was used. Elf3 CRISPR lentiviral particles (Target ID Hs0000109975 Exon2) (SIGMA) were applied to cells as per manufacturer’s instructions. Briefly, cells were infected with lentiviruses, and 48 h later, puromycin 1 µg·mL^−1^ was added. Puromycin selection was applied for 11 days and cells plated at low density. Multiple colonies grew out and were ring‐cloned and expanded. Cells were then pelleted for RNA, DNA and protein. PCR and sequencing was carried out to validate clones after cleaning the PCR product with Qiagen PCR Cleanup Kit. (Primers used: Elf3_Ex1_Crispr_F CAT CCT CTC TCC CCC TAC CC and Elf3_Ex1_Crispr_R TGA GAC CCA CCT GTA CCC TC). CRISPR‐treated clones with various genetic changes, but low/no expression of ELF3, were compared with mock‐infected puromycin‐resistant clones by Affymetrix Clariom D gene expression microarray.

### Statistical analyses

Unless otherwise stated, statistical analyses were carried out using graphpad prism 6/7 software (San Diego, CA, USA). Functional assays were carried out in technical triplicate (at the same time with the same batch of cells) and biological triplicate (at a separate time point with a different batch of cells). Significance was calculated on at least three biological replicates using tests described in figure legends. Results were expressed as the mean with associated standard deviation unless otherwise stated. Statistical significance was represented on graphs as *P = 0.01 to 0.05, **P = 0.001 to 0.01, ***P = 0.0001 to 0.001, ****P < 0.0001.

## Results

### ELF3 is expressed in the committed basal cell population of the prostate

**Fig. 1:**
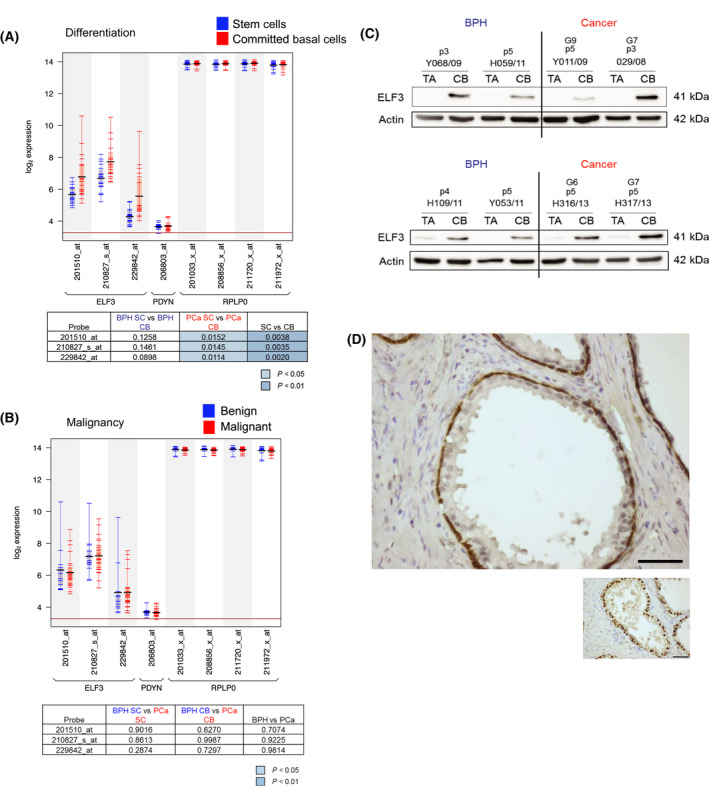
ELF3 is expressed in basal epithelial cells of human prostate. (A, B) Affymetrix gene expression arrays were carried out on the sorted cell populations from BPH (*n* = 7) and PCa (*n* = 12) tissues obtained from patients. ELF3 gene expression was compared between stem cell (SC) and committed basal (CB) cell populations, and between benign and malignant samples, derived from BPH and cancer tissues. RPLPO (ribosomal protein lateral stalk subunit P0) and PDYN (prodynorphin) were used as positive and negative control genes, respectively. Black horizontal bars indicate mean and coloured bars indicate individual patient samples. Tables indicate the *P* values of individual ELF3 probes in SC vs. CB (A) and BPH vs. cancer (B). Statistical significance was measured using the Student’s *T*‐test (unpaired, two‐tailed). Significant values are highlighted in blue. Data generated by mining array from Birnie et al. [31]. (C) Western blot analysis of ELF3 protein expression in the TA and CB cell subpopulations of primary BPH (*n* = 2) and PCa (*n* = 2) samples. Actin was used as a loading control. p = passage number. G = Gleason score of cancer sample. (D) IHC was carried out on formalin‐fixed, paraffin‐embedded BPH tissue using the ImmPRESS Excel Amplified HRP Polymer Staining Kit (Vector Labs) and sections were incubated with DAB and then counterstained with haematoxylin. Sections were stained for ELF3 (Ab97310) and Nkx3.1 (nuclear luminal cell control) – small image. Scale bar = 50 μm.

ELF3 mRNA expression levels were originally measured in an Affymetrix gene expression microarray originally carried out in our laboratory, on enriched stem cell (SC) and committed basal (CB) epithelial cell populations derived from both human BPH and PCa tissue [31]. A total of seven BPH and twelve PCa tissues were processed and enriched for SC (α2β1^hi^/CD133^+^) and CB cells (α2β1^lo^/CD133^‐^). There was a significant increase in *ELF3* gene expression in CB cells compared with SC across three individual probes (Fig. 1A). However, there was no significant difference in ELF3 gene expression between benign and malignant samples when cell types were pooled (Fig. 1B).

At the protein level, initial western blots indicated that lysates containing whole unfractionated populations from patient‐derived primary epithelial cell cultures and lysates from primary stromal cell cultures did not express detectable ELF3, the latter being expected for an epithelial‐specific gene (Fig. S1). BPH and PCa tissues were then cultured and enriched for transit amplifying TA (α2β1^hi^/CD133^‐^) and CB subpopulations. ELF3 was consistently expressed in the CB population of both BPH (*n* = 4) and PCa (*n* = 4) samples (Fig. 1C). There was very little expression or no detection of ELF3 in the TA population of any samples. Stem cells constitute a very small percentage of the TA progenitor cell population (< 0.1%). Furthermore, there was no differential expression between BPH and PCa.

Immunohistochemistry of formalin‐fixed, paraffin‐embedded BPH tissue localised ELF3 staining to the basal layer of the basal/luminal epithelial bilayer in prostate acini (Fig. 1D). Nkx3.1 was used as a control for nuclear staining in luminal epithelial cells (small image in Fig. 1D).

To model ELF3 expression profiles in a panel of prostate cell lines, ELF3 protein expression was measured by western blotting (Fig. S2). Here, ELF3 was most highly expressed in the normal PNT2‐C2 cell line, but was only expressed at relatively low levels in the PNT1a cell line (also derived from normal prostate): both represent androgen‐independent cells. The BPH‐1, PC3 and Du145 cell lines showed moderate expression, whilst P4E6, LNCaP and 22RV1 expressed relatively low levels of ELF3.

### ELF3 knockdown reduces viability, wound healing and colony‐forming ability in both benign and cancerous prostate epithelial cell lines

**Fig. 2:**
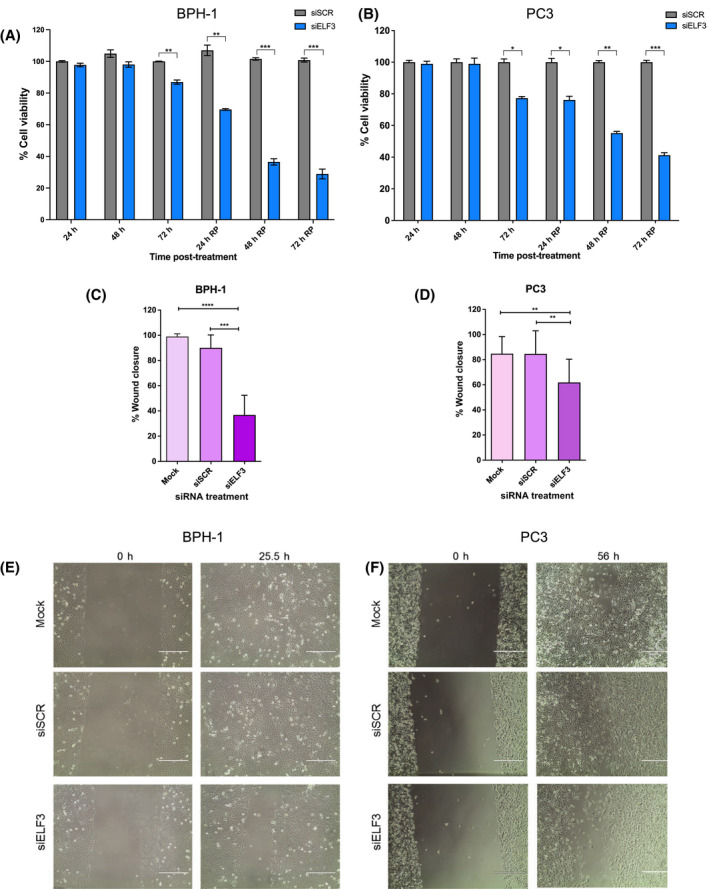
ELF3 knockdown reduces the cell viability and cell migration of benign and cancerous prostate cells. (A, B) AlamarBlue cell viability assays were performed 24 h after transfection and every day for six consecutive days. Cells were replated after the day 3 reading at the original seeding density, (*n* = 3). Mock samples were normalised to 100% viability and siSCR‐ and siELF3‐treated samples plotted relative to mock. Statistical significance was determined using a Student’s *T*‐test comparing siSCR‐treated and siELF3‐treated samples at each time point. (C–F) Migration of BPH‐1 and PC3 cells following knockdown calculated by % wound closure (*n* = 3). Statistical significance was determined using a one‐way ANOVA with Tukey’s correction. Error bars are mean with standard deviation.

As ELF3 was expressed in prostate basal epithelial cells, BPH‐1 and PC3 cells were considered appropriate representative cell lines to initially characterise the effects of *ELF3* knockdown in prostate benign and cancer cells, as they both express significant levels of ELF3 and do not have the characteristics of a luminal cell phenotype. ELF3 siRNA transfection sustained knockdown for at least 6 days post‐transfection (Fig. S3). This ensured that ELF3 expression remained at a very low level for the duration of all the functional experiments that were carried out. In both BPH‐1 and PC3 cells, the viability of cells with *ELF3* knockdown decreased significantly relative to scrambled siRNA‐transfected cells on day 3 with a larger decrease following replating at days 4, 5 and 6 (Fig. 2A,B). In both BPH‐1 and PC3, cells viability had decreased by around 60–70% by day 6. Cell motility was also significantly decreased following *ELF3* knockdown in both BPH‐1 and PC3 cells (Fig. 2C–F). Whilst BPH‐1% wound closure was decreased by around 60% by *ELF3* knockdown, PC3 cells had a more modest decrease of around 20% compared with the scrambled siRNA control.

**Fig. 3:**
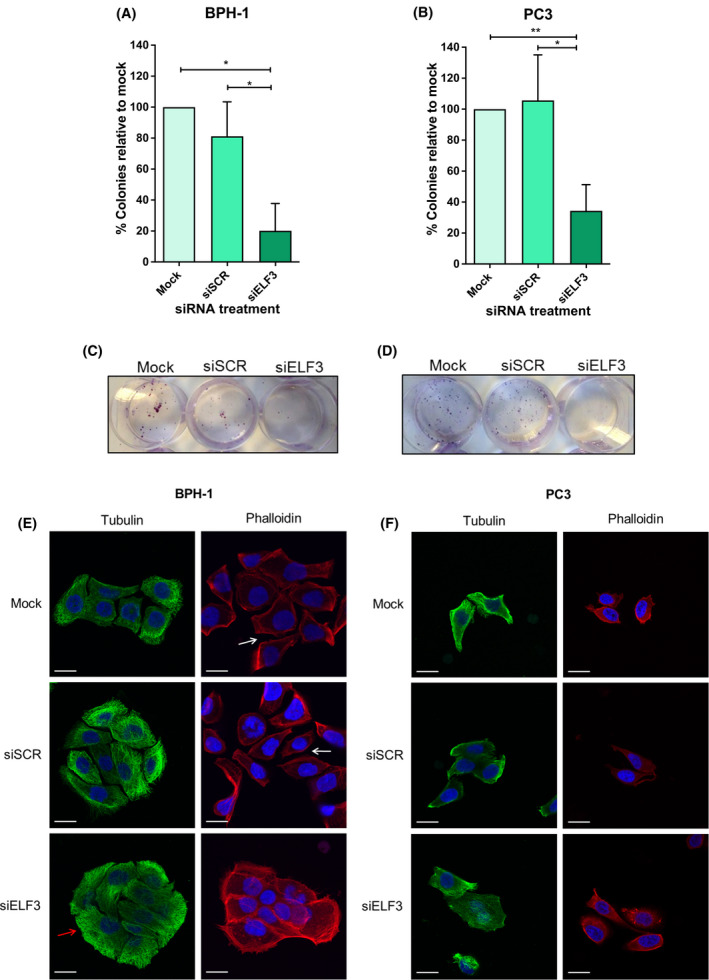
ELF3 knockdown reduces the colony‐forming ability of benign and malignant prostate cells. (A, B) Colony‐forming assays were performed on BPH‐1 cells and PC3 cells 24 h after transfection. Number of colonies > 32 cells were counted. Mock control samples were normalised to 100% and siSCR and siELF3 samples plotted relatie to mock. Statistical significance was determined using a one‐way ANOVA with Tukey’s correction. Error bars are mean with standard deviation. (C, D) Representative images of colonies following crystal violet staining, (*n* = 3). (E, F) BPH‐1 and PC3 cells were transfected with ELF3 (siELF3) and scrambled siRNA (siSCR), fixed in 4% paraformaldehyde at day 3 post‐transfection and stained with tubulin (green) and phalloidin (red), blue = DAPI. Scale bar = 20 μm. Representative colonies shown. White arrows show spaces between cells in mock and siSCR cells. The red arrow highlights the location of the tubulin at the colony periphery.

*ELF3* knockdown significantly decreased the colony‐forming ability of prostate epithelial cell lines (Fig. 3A–D). BPH‐1 cells with *ELF3* knockdown had about 60% decrease in colony formation, whilst PC3 cells had around a 60–70% decrease in colony formation relative to scrambled siRNA‐transfected cells. Morphology of cell colonies was also analysed post‐transfection using tubulin and phalloidin staining (Fig. 3E,F). The colonies of BPH‐1 cells transfected with siELF3 were more compact, with cells closer together, and with higher concentration of tubulin and phalloidin around the edge of the cells on the outside of the colonies. This effect was not as pronounced in PC3 cells perhaps due to the different nature of growth of PC3 cells with less condensed colony formation compared with BPH‐1.

As the most significant decrease in cell viability did not occur until after replating the transfected cells (Fig. 2A,B), it was hypothesised that some cells with *ELF3* knockdown had disrupted adhesion capabilities. An adhesion assay showed that *ELF3* knockdown decreased BPH‐1 cell adhesion regardless of starting density (Fig. S4A). However, as there was a continued decrease in cell viability following day 3, as opposed to an increase which would be evident with normally proliferating cells, this suggested that there was another mechanism contributing to the progressive decrease in viability overtime. To assess whether reduced cell viability had occurred due to cell death by apoptosis, western blot analysis was carried out on BPH‐1 and PC3 cells using cleaved caspase 3 as a marker of apoptosis. BPH‐1 cells treated with 1 μm staurosporine for 24 h were used as a positive control as staurosporine is a non‐selective protein kinase inhibitor that induces apoptosis. Cleaved caspase 3 was only present in staurosporine‐treated cells, and therefore, *ELF3* knockdown cells were not dying via apoptosis (Fig. S4B,C). This correlates with the observation that little cell death was observed by microscopy and suggests the decrease in viable cell number may be due to reduced proliferation.

### ELF3 is a regulator of the cell cycle in prostate epithelial cells

**Fig. 4:**
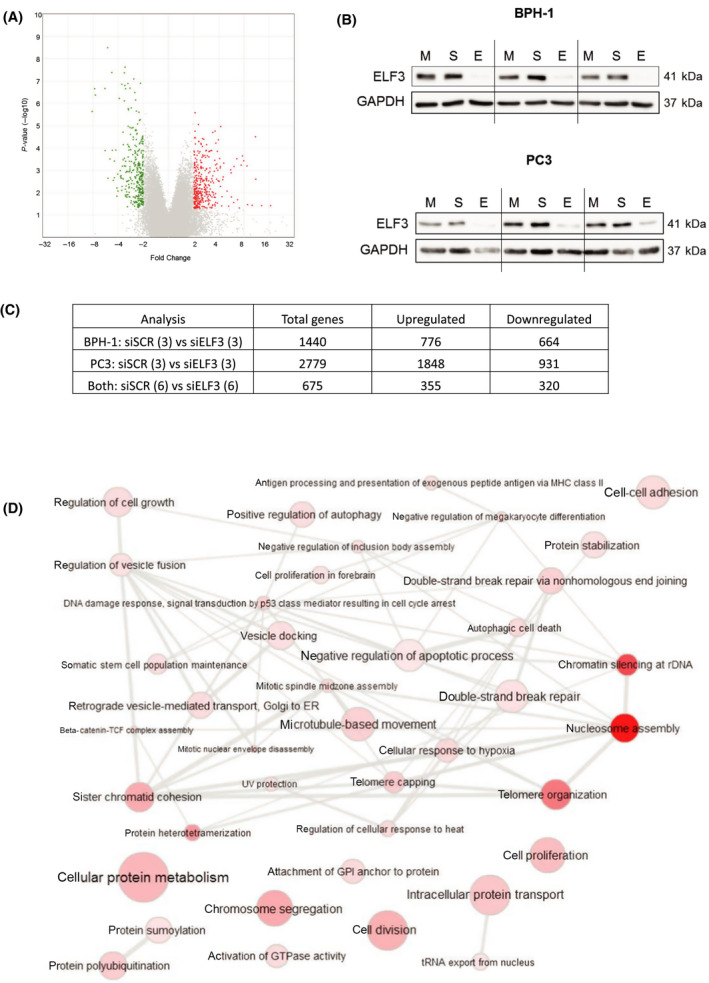
Differential gene expression of BPH‐1 and PC3 cells with ELF3 knockdown was determined by an Affymetrix Clariom D microarray. (A) Volcano plot indicating gene expression changes of siSCR vs. siELF3 BPH‐1 and PC3 cells collectively. Significance threshold included genes with a 2‐fold increase or decrease and a *P*‐value < 0.05. Red = increased expression, green = decreased expression. (B) Western blot indicating ELF3 protein expression 72 h following ELF3 knockdown. GAPDH was used a loading control. (*n* = 3) M = mock, S = siSCR, E = siELF3 (C) RNA extracted from duplicate samples were sent for Affymetrix gene expression microarray. Number of differentially expressed genes in each gene expression microarray analysis. (D) Gene ontology terms associated with significant differential gene expression changes between siSCR and siELF3 cells. Generated via the Database for Annotation, Visualization and Integrated Discovery (DAVID) and visualised using REVIGO.

To investigate potential networks controlled by ELF3, we employed a transcriptomic approach. Sample sets consisted of BPH‐1 and PC3 cells in *n* = 3 biological repeats (siSCR and siELF3). Each set was carried out in duplicate to extract RNA for a gene expression microarray (ArrayExpress accession E‐MTAB‐11485) and also protein for validation of ELF3 knockdown, and to validate differentially expressed genes of interest from the array at the protein level. The differential gene expression between siSCR and siELF3 samples of BPH‐1 and PC3 cells was analysed both individually and collectively with a significance threshold of twofold increase or decrease and a *P*‐value < 0.05 (Fig. 4A). There was a significant downregulation of ELF3 at the protein level in all siELF3‐treated samples (Fig. 4B). PC3 cells had the most appreciable response to *ELF3* knockdown, with a total of 2779 differentially expressed genes (including putative unannotated transcripts), compared with 1440 genes in BPH‐1 cells (Fig. 4C). A total of 675 genes were differentially expressed between siSCR and siELF3 samples in both cell types, implying that ELF3 is involved in similar pathways in both cell lines. Some of the other most affected genes are indicated in Table 1.

**Table 1:** Selection of genes with highest expression change following *ELF3* knockdown.

ETS transcription factors		BPH‐1		PC3		Both	
Gene	ETS subfamily	Fold change	*P*‐value	Fold change	*P*‐value	Fold change	*P*‐value
ESE3	ESE	1.32	3.18E‐02	6.94	7.29E‐06	2.77	0.4516
ELF1	ELF	2.06	0.0345	1.51	0.0549	1.61	0.2949
ETS1	ETS	2.21	4.78E‐05	1.15	0.5584	1.46	0.0851
ETS2	ETS	1.72	0.0287	2.54	0.0002	2.12	0.1448
ETV4	PEA3	−1.45	0.0468	−4.91	4.84E‐05	−2.88	0.1632
Differentiation‐associated genes							
CDH1	E‐cadherin	1.13	0.2709	2.34	0.005	1.79	0.6553
ITGA5	Integrin a5	1.81	0.0935	2.84	0.001	2.33	0.0239
FN1	Fibronectin	−2.58	0.5795	3.64	0.0002	2.86	0.3618
ENO2	Neuron specific enolase	2.6	0.0436	7.21	2.09E‐05	3.53	0.0158
Stem cell markers							
ITGA2	Integrin, alpha 2 (CD49B)	2.89	0.0047	1.48	0.3968	1.7	0.613

Gene Ontology analysis of all data sets revealed multiple terms associated with cell cycle‐related processes and histone‐regulated processes (Fig. 4D). In agreement with this, several of the most altered individual genes in both cell lines following *ELF3* knockdown included cell cycle‐related genes and histone genes. (Table S4 shows expression changes of cell cycle‐related genes following *ELF3* knockdown from gene expression microarray.) Most notably, the serine/threonine protein kinase *PLK1* was downregulated 7.5‐fold in siELF3 samples compared with siSCR control in both BPH‐1 and PC3 cells combined. Furthermore, when BPH‐1 and PC3 cells were analysed in separate analyses, *PLK1* was downregulated 15.5‐fold in BPH‐1 cells and over 5.5‐fold in PC3 cells compared with siSCR control. PLK1 is involved in several stages of the cell cycle, most notably during late G2 and cytokinesis [32, 33]. Other altered genes from the microarray were also linked to G2 cell cycle phase and the PLK1 pathway, such as a decrease in *CDC25C* and increase in *p21* relative to siSCR control.

**Fig. 5:**
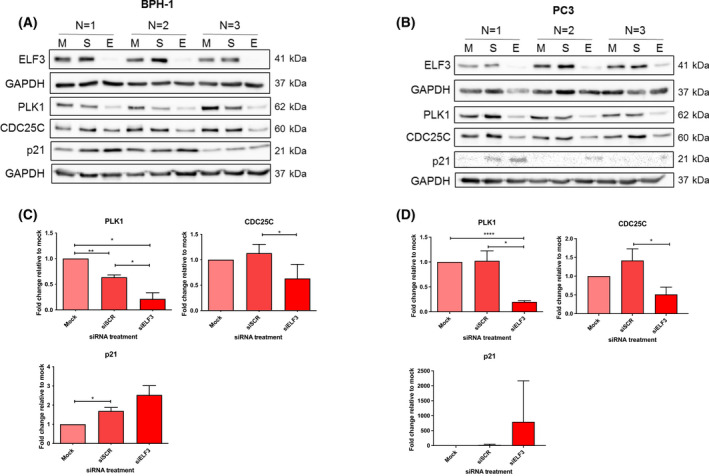
ELF3 knockdown induces changes in the expression of key cell cycle regulators. Representative western blots and densitometry analysis of G2 phase cell cycle‐related genes in (A, C) BPH‐1 cells (*n* = 3) and (B, D) PC3 cells (*n* = 3) 72 h following ELF3 knockdown. M = mock, S = siSCR, E = siELF3. Statistical significance was determined using a one‐way ANOVA with Tukey’s correction. Error bars are mean with standard deviation.

**Fig. 6:**
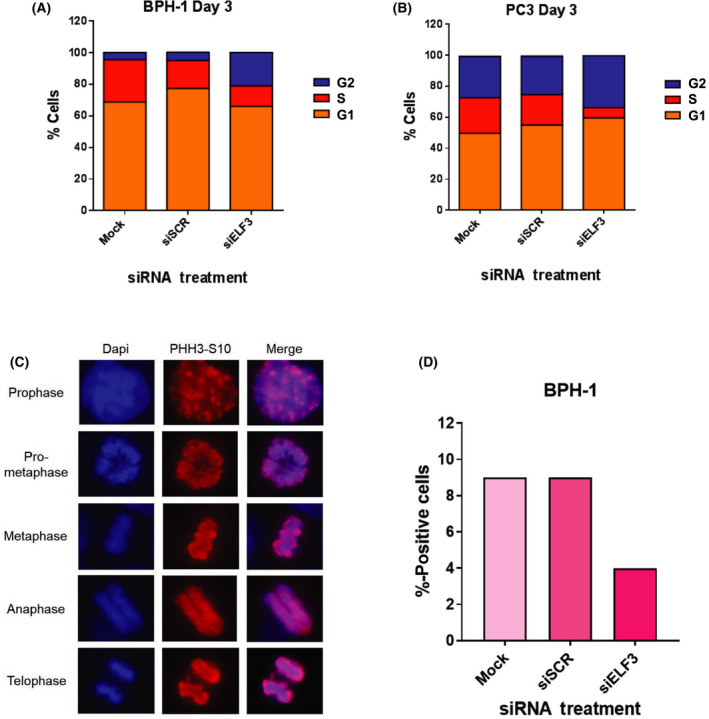
ELF3 knockdown causes a progressive accumulation of cells in the G2 phase of the cell cycle and reduces the number of BPH‐1 cells in mitosis. Cell cycle analysis was carried out on (A) BPH‐1 and (B) PC3 cells 72 h post‐transfection by detecting EdU and propidium iodide. Graphs represent the mean of biological triplicates (*n* = 3). (C, D) BPH‐1 cells were transfected with siELF3, fixed in 4% paraformaldehyde at day 3 post‐transfection and stained with phospho‐histone 3 (PHH3‐S10) as a marker of mitosis. Blue = DAPI, red = PHH3‐S10. Representative images of cells in each stage of mitosis are shown. 220 cells were counted in each condition and presented as a per cent PHH3‐S10‐positive.

Several G2 phase‐associated proteins of interest were validated by western blot using lysates from the BPH‐1 and PC3 siELF3 cells (Fig. 5A–D). *PLK1* and *CDC25C* were downregulated in both PC3 and BPH‐1 cells at the protein level relative to controls. Expression levels of the cyclin‐dependent kinase (CDK) inhibitor *p21* were increased relative to controls. These changes correlated with an arrest at the G2 checkpoint which was confirmed by a progressive accumulation of cells in the G2 phase during cell cycle analysis with reduction of cells in S phase (Fig. 6A,B). Phosphohistone H3 (PHH3) ICC staining was also carried out on BPH‐1 cells to determine the number of cells in mitosis with siELF3 compared with siSCR. Representative images indicating the different stages of mitosis are shown (Fig. 6C). Whilst 9% of mock and siSCR cells were positive for PHH3, only 1.8% of siELF3 cells were undergoing mitosis, further indicating G2 cell cycle arrest (Fig. 6D). The absence of ELF3 leading to cell cycle arrest is indicative of ELF3 acting as a cell cycle regulator and promoter of cell cycle.

### Expression of ELF3 in prostate cancer tissue is variable

**Fig. 7:**
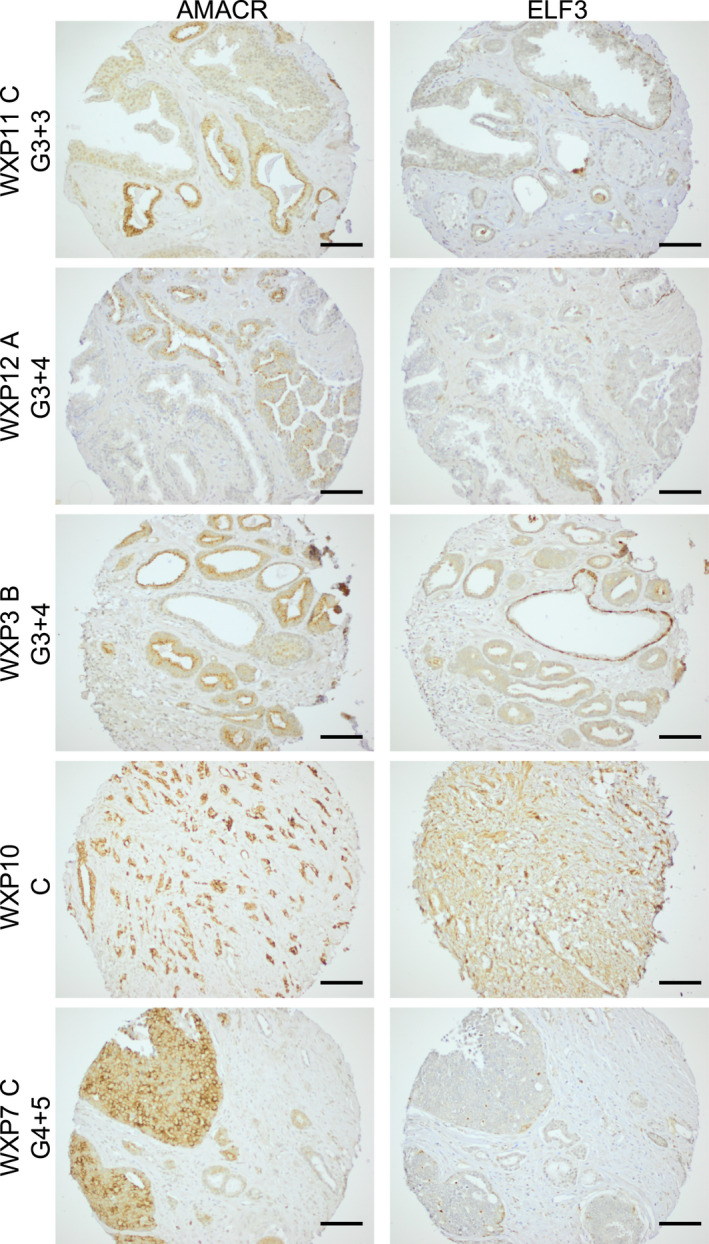
ELF3 expression in Cancer tissue microarrays (TMAs). 40 tissue sections from 13 patients were stained for ELF3 expression using antibody Ab97310 using the Vector ImPRESS Excel Kit. Representative images from several patients are shown; Patient WXP11C (Gleason grade 3 + 3), WXP12A (Gleason grade 3 + 4), Patient WXP3B (Gleason grade 3 + 4), Patient WXP10C (Gleason grade 4 + 3), Patient WXP7C (Gleason grade 4 + 5). Sections were stained for AMACR as a cancer marker. Negative control was used where tissues were stained with secondary antibody only (not shown). Scale bar = 100 μm. A summary of comparative AMACR and ELF3 staining in all 13 PCa patient samples is shown in Table 2 (TMAs provided through collaboration with Professor Dan Berney, Barts and The London School of Medicine and Dentistry).

To relate the *in vitro* studies to whole tissues, expression of ELF3 in benign and malignant patient tissue samples, tissue microarrays were obtained from the Orchid tissue bank (in collaboration with Professor Dan Berney, Barts and The London School of Medicine and Dentistry). BPH tissue (102 sections from 34 patients) and PCa tissue from a range of Gleason grades (40 sections from 13 patients) were stained and analysed. Of the BPH tissue sections from 34 patients, 7 patients only showed stroma, and so were negative for ELF3 protein expression. The sections from the other 27 patients contained epithelial glands and 100% of these were positive for ELF3 expression. Some patient sections showed exclusively cytoplasmic staining whilst others also exhibited nuclear ELF3 (Fig. S5). Results were less clear in PCa tissue (Fig. 7). Loss of the basal cell population and expression of AMACR are indicators of cancer used in prostate histology and contribute to a cancer diagnosis [34]. AMACR (alpha‐methylacyl‐CoA racemase) is an enzyme that is overexpressed in PCa and was therefore used to distinguish areas of cancer in the PCa tissue sections [35]. A summary of comparative AMACR and ELF3 staining in all 13 PCa patient samples is shown (Table 2). Sections of Gleason 6 grade PCa which contained no obvious glandular structures did not express ELF3. However, some sections contained glandular structures both with and without AMACR staining (WXP11C). ELF3‐positive staining was observed in Gleason 7(3 + 4) and Gleason 7(4 + 3) samples, with some quite strong staining, although not in all sections. Lower ELF3 staining was also observed in a proportion of higher Gleason grade (8/9) samples. ELF3 was detected in basal‐like cells of AMACR‐negative glands. In less differentiated sections, with Gleason score ≥ 7, which had no obvious glandular structures, but were entirely AMACR positive, some patients did not stain for ELF3 (WXP7C), whilst others showed stronger ELF3 staining (patient WXP10C). These results suggest that ELF3 expression may initially be repressed in areas of lower grade (Gleason 6) tumours. Additionally, there may be a subset of higher grade prostate tumours (Gleason ≥ 7) which then re‐express ELF3. Further work on additional samples is needed to establish if a correlation between ELF3 protein expression and Gleason score exists.

**Table 2:** Gleason score, AMACR positivity and ELF3 positivity of patient tissue samples on TMA.

Patient	Gleason score	AMACR positivity	ELF3 positivity
WXP11	3 + 3	++	+
		++	+
WXP12	3 + 3, 3 + 4	++	+
		++	−
		++	−
WXP13	3 + 4	+	−
		−	+
		−	−
WXP3	3 + 4	++	++
		++	+
WXP5	3 + 4	++	+++
WXP9	3 + 4	−	−
		−	−
		−	−
8929‐02	4 + 3	++	−
		+++	−
		+++	+++
WXP6	4 + 3	++	+
		+++	−
		+++	+
WXP8	3 + 4, 4 + 3	+++	+
		++	+
		+++	+
WXP10	4 + 3	+++	+
		+++	+++
WXP2	4 + 4	+	−
		+	−
		+	−
WXP4	4 + 4	++	+
		−	+
WXP7	4 + 4, 4 + 5	+++	−
		+++	+
		+++	+

### Amplification of ELF3 is observed in metastatic prostate cancer

**Fig. 8:**
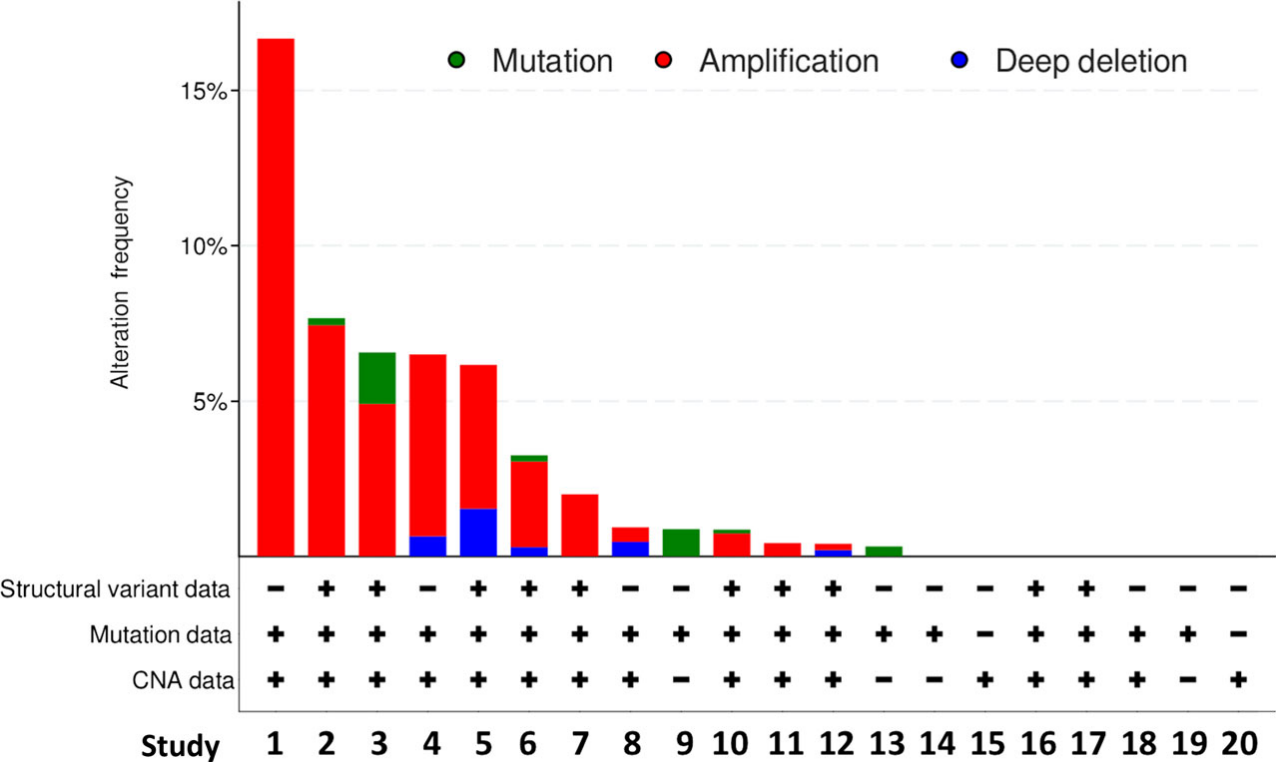
ELF3 genetic alterations in prostate cancer. Twenty published studies were analysed for genetic alterations of ELF3 in PCa [8, 61, 62, 63, 64, 65, 66, 67, 68, 69, 70, 71, 72, 73, 74, 75, 76, 77, 78]. These were classified into amplification, mutation, deletion and multiple alterations. The studies analysed a range of 12–1465 patients (6329 samples from 6044 patients). The most common ELF3 genetic alteration was amplification and ELF3 genetic alterations were mostly observed in metastatic PCas (Studies detailed in Table S5).

To further examine the activation profile of ELF3 in PCa, *ELF3* genetic alterations were investigated in publicly available data sets using cBioPortal [36, 37] (Fig. 8). Whilst there were few or no alterations found in data sets consisting of mainly primary localised PCas, those data sets that included metastatic PCas showed a subset of patients with genetic alterations of *ELF3*, including mutations and deletions but most commonly amplification. These findings suggest that *ELF3* amplification is associated with late stage disease and may provide a survival benefit to a subset of more advanced cancers.

### Upregulation of ELF3 by Vorinostat

Histone deacetylase (HDAC) inhibitors have been shown to reduce AR expression and to induce a neuroendocrine (NE) phenotype in some prostate cells [38, 39, 40]. To determine whether ELF3 was associated with a NE phenotype in PCa, we treated primary prostate epithelial cultures derived from tumours with the HDAC inhibitor vorinostat. Vorinostat upregulated ELF3 in a concentration and time‐dependent manner in TA, CB and whole populations of basal epithelial cells (Fig. S6). In addition, increased vorinostat did lead to a morphology change in the cells.

### CRISPR knockout of ELF3 in BPH1 cells

As ELF3 inhibition by siRNA can function to block cell proliferation and to lower clonogenic potential, it would seem to be a good candidate for small molecule inhibition. However, the transient nature of siRNA inhibition provides few clues about the longer‐term responses of cells to loss of ELF3 expression. As with all strategies to block ETS factors, there is always the possibility that there would be redundancy and an ‘escape’ mechanism involving the activation of another ETS family members. To test this hypothesis, a CRISPR knockout of ELF3 was carried out (Fig. S7). Perhaps surprisingly, given the effects of siRNA, viable proliferating colonies were derived, and of the four clones (showing no ELF3 expression) analysed, two clones (insertion and multiple edits) showed increase of p21 and the two deletion clones showed no change in p21; however, PLK1 expression was increased in all four clones studied, rather than decreased as was observed following siRNA knockdown. An aggregated gene expression microarray analysis of 10 such ELF3 deleted BPH1 clones identified changes in the expression of five ETS family members: *ELF5*(−3.2); *ERF*(−2.1); *ETV4*(2.5); *ETV5*(6.6); *ETV7*(−3.1). Values in brackets indicate fold changes. Most striking was the increased *ETV5* expression, which is in contrast to the result in the siRNA knockdowns in BPH‐1 cells where ETV5 expression decreased: *ELF5*(−1.11); *ERF*(−1.27); *ETV4*(−1.45); *ETV5*(−1.36); *ETV7*(1.49).

## Discussion

Deregulation of the ETS transcription factor family members is now recognised as a common feature in multiple cancers; with aberrant expression, loss of tumour suppressor function, inactivating mutations and the formation of fusion genes all observed [41]. Furthermore, these ETS factors also play a role in epithelial cell differentiation and consequently may be important regulators of the SC (and cancer SC) phenotype in the prostate [42].

Neither the Longoni study [19] nor the Shatnawi study [12] used normal or benign cells as comparison controls in the biological studies, and each study used different cancer cell models; thus, conclusions were that ELF3 might be a tumour suppressor or oncogene, or it may be context and cell‐dependent. Therefore, the study presented here used alternative cell line models (BPH‐1 and PC3 cells), based on their expression levels of ELF3, and patient‐derived primary prostate epithelial cells and patient tissue microarrays to further elucidate the role of ELF3 in both normal prostate growth and PCa.

A key factor to identify the function of ELF3 was to consider the heterogeneous cell populations present in the prostate gland. This work built on a gene expression microarray that was previously carried out to assess the differential gene expression between prostate SCs and the more differentiated CB cells derived from benign and cancerous prostate tissue [31]. ELF3 was identified as significantly upregulated in the more differentiated CB cells of the prostate epithelium compared with SCs, irrespective of diagnosis. Expanding on this, this study confirmed that ELF3 was predominantly expressed in prostate basal epithelial cells (CB) and also showed it was not expressed in early progenitor cells (TA), luminal epithelial cells or stroma. Examination of ELF3 protein expression was carried out in prostate tissue sections of BPH (showing defined glands) but also by measuring ELF3 expression in TA and CB cells enriched from cultured patient‐derived primary prostate epithelial cells.

Previous studies have highlighted ELF3 as a cell cycle regulator in breast and non‐small cell lung carcinoma (NSCLC) cell lines. Analysis presented here showed that silencing ELF3 resulted in decreased viability, reduced cell migration and decreased colony formation along with morphological changes in colony shape with no evidence of apoptosis. A global analysis of the effect of silencing ELF3 was carried out by microarray analysis, and GO analysis from the microarray revealed that the differentially expressed genes were involved in processes related to the cell cycle and histone regulation (Table S4).

*PLK1* was one of the most differentially expressed genes and was amongst the highest fold changes in both analyses (BPH‐1, PC3). PLK1 is involved in both late G2 and cytokinesis stages of the cell cycle, both of which were highlighted in the GO analysis, [32, 33]. Histone gene expression is highly regulated during the cell cycle, which may account for the bias towards histone‐regulated processes in the GO analysis. There were also changes in the expression of genes upstream and downstream of the PLK1 pathway, including *CDC25C*, *cyclin B1*, *p21* and *FOXM1*. Of note, increased PLK1 expression has been independently detected in PCa [43], and there is strong evidence for a functional role for FOXM1 in promoting PCa cell proliferation both in vitro and in vivo and in patient tissues [44, 45]. There was a decrease in PLK1 and CDC25C as well as an increase in CDK inhibitor p21 after ELF3 knockdown. This was also validated at the functional level, where there was an accumulation of BPH‐1 and PC3 siELF3 cells in the G2 phase overtime and a reduced number of cells in S phase. Furthermore, PHH3‐S10 staining confirmed there were fewer siELF3 cells undergoing mitosis compared with mock and siSCR. This conclusively validates ELF3 as a cell cycle regulator in prostate epithelial cell lines regardless of diagnosis.

Whilst the variation in response to the CRISPR knockouts compared with the siRNA knockdown could indicate off‐target effects with either, it could equally be indicative of an alternative mechanism at play due to the difference between the two methods. The absence of ELF3 expression after siRNA treatment was shown to cause cell cycle arrest, which would result in no or reduced viable colony formation. However, multiple viable clones *were* retrieved, and therefore, the explanation for the disparity is most likely to be a salvage pathway in the CRISPR clones, resulting in alternative activation of PLK1. When the expression of other ETS factors in both the siRNA knockdowns and the CRISPR knockouts was measured by Gene array anaylsis, we found that after siRNA ELF3 knockdown, ETV5 expression also decreased, perhaps indicative of a co‐regulatory process. However, in the CRISPR ELF3 knockouts, an increase in *ETV5* expression was observed. Just such an ELF3/ETV5 compensation was identified in knockout mice with homozygous deletion of ELF3 (*Elf3‐/‐*). The mice were viable and in the lungs of adults, where ELF3 is required for epithelial integrity, ETV5 was highly expressed in epithelial cells [46]. Such a compensation mechanism could render the efficacy of drug‐targeted ELF3 inhibition ineffective for longer‐term treatment of cancer cells. Furthermore, overexpression of ETV5 has been correlated with higher malignancy in multiple human tumour types [47] including PCa, where it is present in a fusion with the TMPRSS2 gene [48].

When the number of genes whose expression was altered by ELF3, siRNA knockdowns was analysed, the cancer cells (PC3) possessed almost double the number of significantly altered genes (1848 upregulated, 931 downregulated) compared with BPH‐1 (776 upregulated, 664 downregulated). Despite more expression alterations, PC3 cells showed a more modest inhibition of colony forming and migration assays compared with BPH‐1 cells, following siELF3 knockdown. Given that the degree of knockdown was similar in both cell lines, this result implies that advanced grade PC3 metastatic cancer‐derived cells may already have upregulated compensatory pathways and alternative mechanisms to cope with *ELF3* knockdown.

Analysis of benign prostate tissue tissue microarrays showed that ELF3 protein was expressed in all tissue sections that contained epithelial glands, confirming the pattern of expression seen previously in individual patient sections. Many cancer sections either showed no ELF3 staining, or showed high ELF3 expression in non‐cancerous, AMACR‐negative glands, regardless of Gleason grade. Some more advanced, less differentiated cancers of Gleason grade ≥ 7 were positive for ELF3 expression. Longoni et al. showed that a subset of prostate tumours expressed higher ELF3 than normal prostate by qRT‐PCR and IHC [13]. However, there was no further distinction between Gleason grades in the latter study. Bioinformatic analysis of two independent data sets also found increased ELF3 expression in metastatic tumours compared with primary prostate tumours, indicating that ELF3 expression may be associated with more advanced tumours [13]. This was also highlighted by our cBioportal analysis (Fig. 8), which aggregated the results from 20 studies and showed some correlation between ELF3 gene amplification and advanced PCas. Results from other tissues vary; in the colon, liver and lung, ELF3 expression appears to be associated with cancer progression and metastases [49, 50, 51, 52]. In contrast, in oral and ovarian tissue, the development of cancer is associated with a loss of ELF3 expression [53, 54].

The regulatory potential of ETS factors most likely explains the conflicting findings in the literature of ELF3 biological functions such as tumour suppressor vs. oncogene. One explanation for the results showing ELF3 as a repressor is that the experiments were carried out in AR‐responsive LNCaP cells and it is very likely that the advanced cancers where there is amplification of ELF3 would be castration‐resistant tumours where androgen is no longer the driver [12]. This could also explain the variation in tumour immunostaining where some tumours are ELF3 negative and some are positive; there could be a correlation with the presence or the absence of AR, as ELF3 is known to act as a repressor of AR. Future studies might also consider the subset of neuroendocrine (NE) prostate neoplasms which include carcinoid tumours, small cell carcinomas and large cell carcinomas. These classes of prostate tumours are generally very rare and are associated with poor prognosis [55]. Several studies have shown that NE differentiation also occurs in conventional prostate adenocarcinomas, in association with more advanced disease. In particular, an increase in NE cells has been observed following ADT and has been proposed as a mechanism of treatment resistance and therefore linked to the emergence of castration‐resistant prostate cancer (CRPC) [56, 57, 58, 59]. Treatment of primary cells with vorinostat increased ELF3 expression, indicating that ELF3 is epigenetically silenced in TA cells. Whilst the NE cell marker NSE increased with vorinostat treatment in one sample, this induction was not consistent in all samples tested. However, there was a distinct change in cell morphology following treatment, with the presence of dendritic‐like processes characteristic of NE cells [60]. This is reminiscent of a process called NE transdifferentiation, whereby prostate epithelial adenocarcinoma cells acquire NE properties. It could therefore be of interest to investigate any relationship of ELF3 to NE PCas.

The implied redundancy found between ETS factors adds an additional layer of complexity when studying these proteins. Studies in ELF3 and other discussed ETS factors have previously shown that function is extremely context‐dependent, with regard to tissue type, cell type and pathology. Consequently, defining the correct cell type to study ELF3 expression was integral to determine its genuine function in the prostate.

Collectively, the findings presented here suggest ELF3 can act as an oncogene in the PCa setting. However, given that ELF3 is also a regulator of the cell cycle and its expression is associated with differentiation status in normal prostate epithelial cells, this role is also worthy of future investigation to fully elucidate the mechanism of action of the ETS transcription factors. In terms of targeting the proliferation controlled by ELF3 therapeutically, our data would suggest extreme caution as compensatory activation of other ETS family members, most notably ETV5, which is a known oncogene in PCa, could result in cancer progression rather than regression.

## Conflict of interest

The authors declare no conflict of interest. The funders had no role in the design of the study; in the collection, analyses, or interpretation of data; in the writing of the manuscript, or in the decision to publish the results.

## Author contributions

Conceptualization, LKA and NJM; methodology, LKA, NJM and FMF; software, APD and LKA; validation, LKA, HFW, SK and GLKM; formal analysis, LKA and APD; resources, VMM, MSS and DMB; data curation, LKA; writing—original draft preparation, LKA; writing—review and editing, FMF and NJM; supervision, NJM and FMF; project administration, HFW; funding acquisition, NJM. All authors have read and agreed to the published version of the manuscript.

## Supporting information


**Fig. S1**. Protein expression of ELF3 in prostate tissue homogenates and stromal cells. Western blot analysis of ELF3 expression in lysates derived from (a) BPH tissue homogenates (n=7) and (b) enriched stromal cells cultured from tissue (n=6). GAPDH was used as a loading control. Tables show patient details for each corresponding lane. HFF = human foreskin fibroblast cell line. G = Gleason score.
**Fig. S2**. Protein expression of ELF3 in prostate epithelial cell lines. Expression of ELF3 was examined in a range of prostate cell lines at the protein level. Protein expression was analysed by Western blot analysis. 20µg of protein was loaded per lane onto a 10% SDS gel, transferred onto a PVDF membrane and probed for the indicated proteins. Tubulin was used as a loading control. The origin and phenotypic characteristics of prostate epithelial cell lines is also shown [71‐80]. (CK = cytokeratin, AR = androgen receptor, PSA = prostate specific antigen, BPH = benign prostatic hyperplasia, PCa = prostate cancer. Markers of basal cells – CD44, CK5. Markers of luminal cells – CK8, CK18, AR. Expression of CK5 in absence of CK14 indicated an intermediate phenotype.)
**Fig. S3**. Time course of ELF3 knockdown in benign (BPH‐1) and cancer (PC3) prostate epithelial cell lines. ELF3 protein expression was analysed by Western blot in (a) BPH‐1 and (b) PC3 cells following ELF3 knockdown over a 6 day time course (n=1 each day / n=6 over 6 days). GAPDH was used as a loading control. Densitometry was carried out using Image J software. Numbers below blots indicate levels of knockdown compared to samples treated with siSCR on the same day. siSCR samples were normalised to 1.0. M = Mock, S = siSCR, E = siELF3. (c) Charts show (i) range of densitometry values comparing paired siSCR and siELF3 treated cells and also showing (ii) range of densitometry values across six days. One‐way ANOVA with Tukey’s correction was used to compare the samples. Error bars are standard deviation of the mean.
**Fig. S4**. ELF3 knockdown reduces cell adhesion but does not cause cell death via apoptosis. (a) An adhesion assay was performed on BPH‐1 cells 48h following knockdown (n=3). Cells were trypsinised and re‐plated at three different densities for 4 hours (High = 300,000 cells, Med = 100,000 cells, Low = 40,000 cells). Floating cells were washed off and adherent cells were counted using a cell counter. Statistical significance was determined using a Student’s T‐test (unpaired, two‐tailed). Error bars are standard deviation of the mean. To assess cell death by apoptosis, lysates of (b) BPH‐1 (n=2) and (c) PC3 (n=2) cells with ELF3 knockdown were probed for cleaved caspase 3. BPH‐1 cells treated with 1μM Staurosporine for 24h were used as a positive control for apoptosis (+).
**Fig. S5**. ELF3 Expression in BPH tissue. (a) ELF3 expression was analysed in BPH tissue by immunohistochemistry (immunofluorescence). Formalin‐fixed, paraffin‐embedded BPH tissue was deparaffinised and rehydrated before undergoing heat‐inducing antigen retrieval in sodium citrate buffer. IHC was carried out and sections were incubated with fluorescent Alexa Fluor secondary antibodies. (i) ELF3 alone (Ab97310), (ii) ELF3 co‐stained with high molecular weight cytokeratin (cytoplasmic basal cell marker) and (iii) ELF3 co‐stained with p63 (nuclear basal cell marker. Red = ELF3, Green = HMW‐CK / p63, Blue = DAPI. 60x oil lens. Scale bar = 10μm. (b) ELF3 expression was analyed in BPH tissue microarrays (TMAs). 102 tissue sections from 40 patients were stained for ELF3 expression using Ab97310 using the Vector ImPRESS Excel Kit. Representative images of sections from two patients are shown. (i) Patient 9C exhibits more cytoplasmic staining whilst (ii) Patient 5A exhibits more nuclear staining. Sections were stained for Nkx3.1 as a nuclear luminal cell control. Sec only = tissues stained with secondary antibody only. 20x scale bar = 100μm, 40x scale bar = 50μm.
**Fig. S6**. ELF3 expression is upregulated in primary prostate epithelial cells following vorinostat treatment. (a) Primary prostate epithelial cell cultures were treated with three different doses of HDAC inhibitor vorinostat (L = 0.625μM, M = 2.5μM and H = 10μM). Cells were harvested at 4 hours and 24 hours after treatment for protein analysis. Numbers below blots indicate expression of ELF3 and NSE relative to the low dose (L) which was normalised to 1. (b) The TA and CB enriched subpopulations were treated with 2.5μM vorinostat for 24 hours before harvesting for protein analysis. WP = whole population. Numbers below blots indicate expression of ELF3 and NSE normalised to the low dose, 4 hour vorinostat treatment. (c) Brightfield images of sample H598/17 24 hours after treatment. Scale bar = 100μm.
**Fig. S7**. Impact of ELF3 CRISPR knockout on cell cycle markers. ELF3 was knocked down in BPH‐1 cells using a CRISPR lentivirus. (a) Four clones were examined for type of genetic alteration that occurred (b) Protein expression levels for ELF3, p21 and PLK1 were measured in each clone using Western Blot analysis. (c) Intensity of bands on Western blot was analysed to determine change in expression of ELF3, p21 and PLK1 in ELF3 clones.
**Table S1**. Antibodies used for protein detection by western blot.
**Table S2**. Antibodies used for protein detection by Immunohistochemistry.
**Table S3**. Antibodies used for protein detection by Immunocytochemistry.
**Table S4**. Expression changes of cell cycle‐related genes following ELF3 knockdown from gene expression microarray. Highlighted boxes indicate genes with significance threshold of 2‐fold increase or decrease and a p‐value <0.05. NS = not significant. (ANOVA analysis with eBayes correction was used).
**Table S5**. List of studies used in Figure 8.

## Data Availability

Microarray data that support the findings in this study are openly available in the ArrayExpress database (https://www.ebi.ac.uk/arrayexpress/) under accession number [E‐MTAB‐11485].
