# Supplementary material for: ETS transcription factor ELF3 (ESE‐1) is a cell cycle regulator in benign and malignant prostate
Source: FEBS Open Bio. 2022 May 6;12(7):1365–87. doi: 10.1002/2211-5463.13417 (PMC9249341; doi:10.1002/2211-5463.13417)
Supplement: Supplementary file 1 — Fig. S1. Protein expression of ELF3 in prostate tissue homogenates and stromal cells. Western blot analysis of ELF3 expression in lysates derived from (a) BPH tissue homogenates (n=7) and (b) enriched stromal cells cultured from tissue (n=6). GAPDH was used as a loading control. Tables show patient details for each corresponding lane. HFF = human foreskin fibroblast cell line. G = Gleason score. Fig. S2. Protein expression of ELF3 in prostate epithelial cell lines. Expression of ELF3 was examined in a range of prostate cell lines at the protein level. Protein expression was analysed by Western blot analysis. 20µg of protein was loaded per lane onto a 10% SDS gel, transferred onto a PVDF membrane and probed for the indicated proteins. Tubulin was used as a loading control. The origin and phenotypic characteristics of prostate epithelial cell lines is also shown [71‐80]. (CK = cytokeratin, AR = androgen receptor, PSA = prostate specific antigen, BPH = benign prostatic hyperplasia, PCa = prostate cancer. Markers of basal cells – CD44, CK5. Markers of luminal cells – CK8, CK18, AR. Expression of CK5 in absence of CK14 indicated an intermediate phenotype.) Fig. S3. Time course of ELF3 knockdown in benign (BPH‐1) and cancer (PC3) prostate epithelial cell lines. ELF3 protein expression was analysed by Western blot in (a) BPH‐1 and (b) PC3 cells following ELF3 knockdown over a 6 day time course (n=1 each day / n=6 over 6 days). GAPDH was used as a loading control. Densitometry was carried out using Image J software. Numbers below blots indicate levels of knockdown compared to samples treated with siSCR on the same day. siSCR samples were normalised to 1.0. M = Mock, S = siSCR, E = siELF3. (c) Charts show (i) range of densitometry values comparing paired siSCR and siELF3 treated cells and also showing (ii) range of densitometry values across six days. One‐way ANOVA with Tukey’s correction was used to compare the samples. Error bars are standard deviation of the mean. Fig. S4 [file FEB4-12-1365-s001.docx]

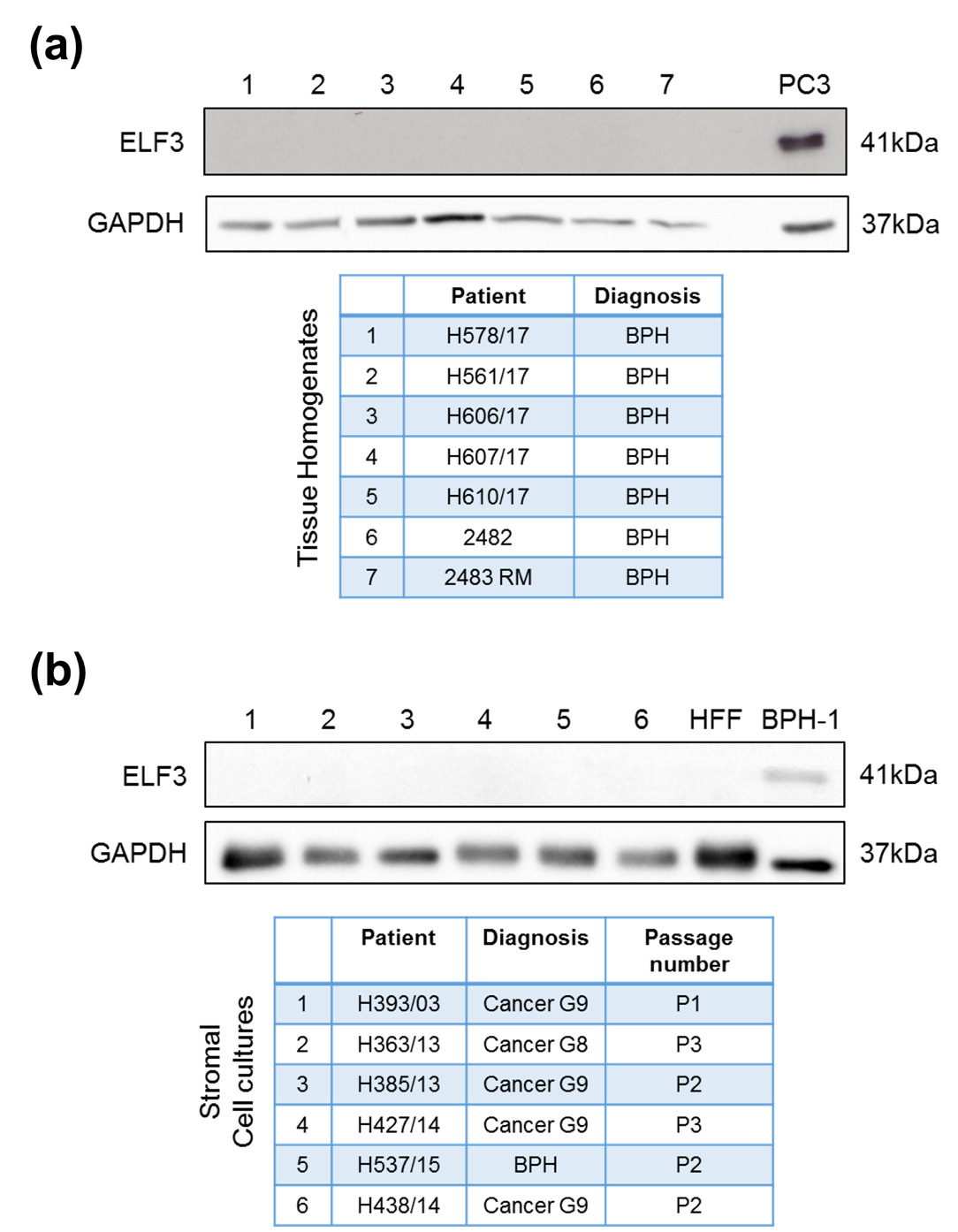


**Supplementary Figure 1. Protein expression of ELF3 in prostate tissue homogenates and stromal cells.** Western blot analysis of ELF3 expression in lysates derived from (a) BPH tissue homogenates (n=7) and (b) enriched stromal cells cultured from tissue (n=6). GAPDH was used as a loading control. Tables show patient details for each corresponding lane. HFF = human foreskin fibroblast cell line. G = Gleason score.


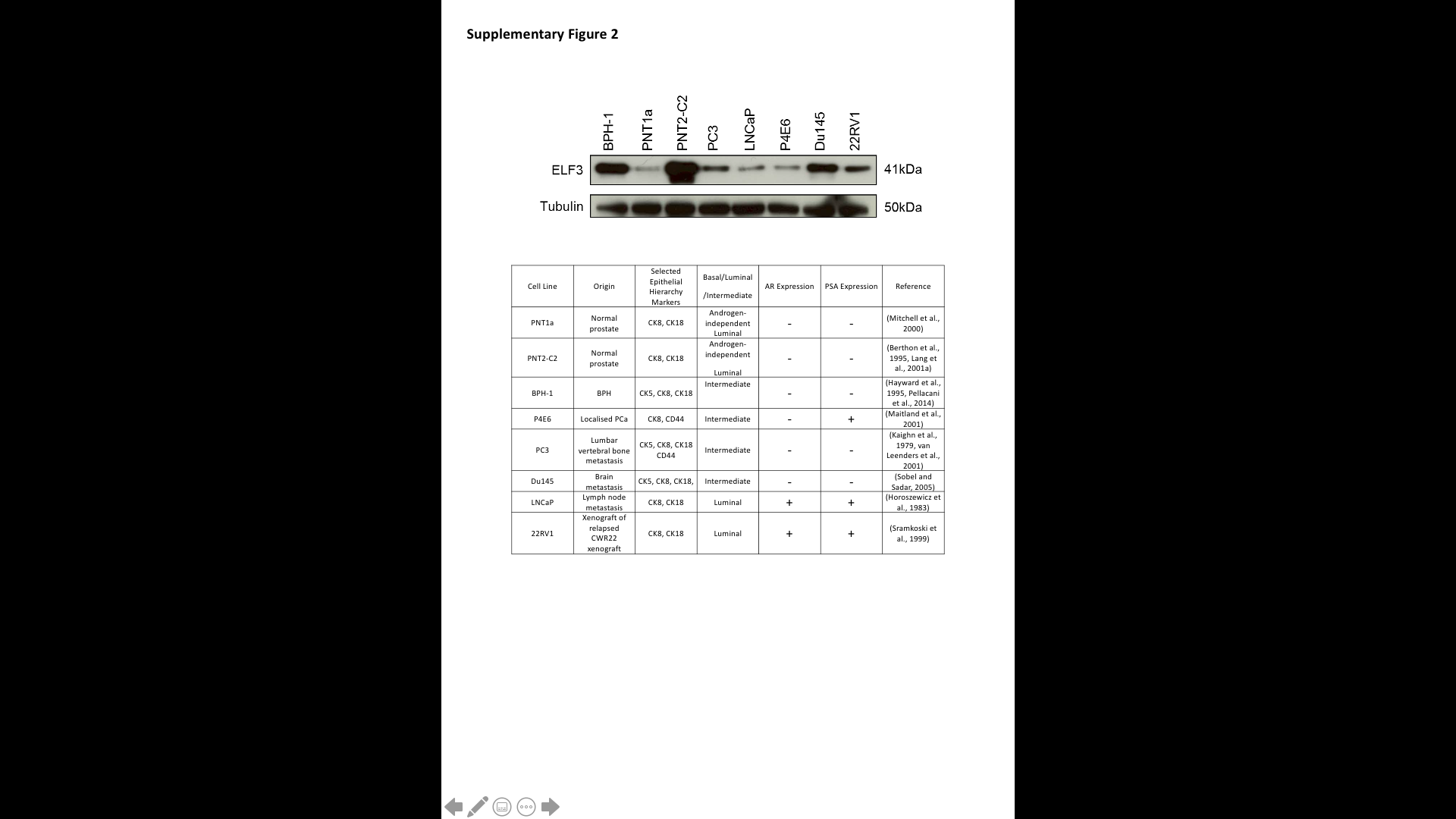


**Supplementary Figure 2. Protein expression of ELF3 in prostate epithelial cell lines.**

Expression of ELF3 was examined in a range of prostate cell lines at the protein level. Protein expression was analysed by Western blot analysis. 20µg of protein was loaded per lane onto a 10% SDS gel, transferred onto a PVDF membrane and probed for the indicated proteins. Tubulin was used as a loading control. The origin and phenotypic characteristics of prostate epithelial cell lines is also shown [71-80]. (CK = cytokeratin, AR = androgen receptor, PSA = prostate specific antigen, BPH = benign prostatic hyperplasia, PCa = prostate cancer. Markers of basal cells – CD44, CK5. Markers of luminal cells – CK8, CK18, AR. Expression of CK5 in absence of CK14 indicated an intermediate phenotype.)


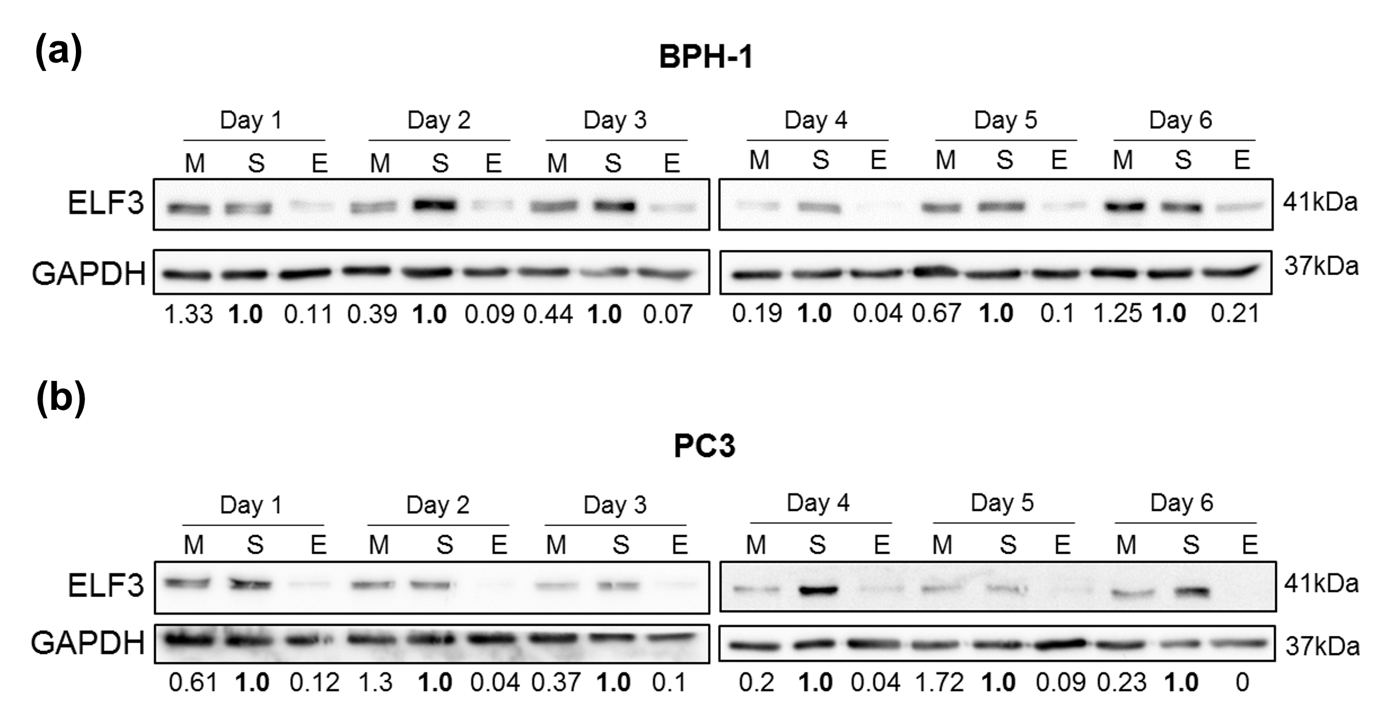


**
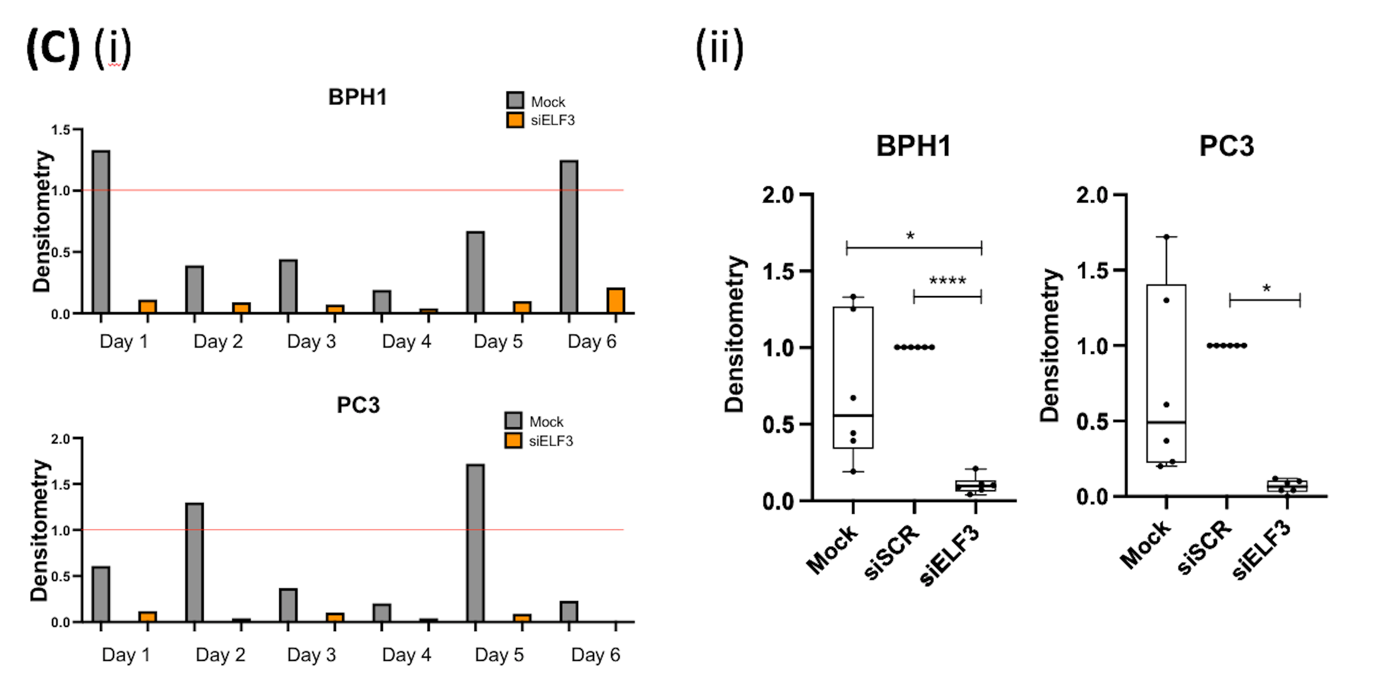
**

**Supplementary Figure 3.** **Time course of ELF3 knockdown in benign (BPH-1) and cancer (PC3) prostate epithelial cell lines.** ELF3 protein expression was analysed by Western blot in (a) BPH-1 and (b) PC3 cells following ELF3 knockdown over a 6 day time course (n=1 each day / n=6 over 6 days). GAPDH was used as a loading control. Densitometry was carried out using Image J software. Numbers below blots indicate levels of knockdown compared to samples treated with siSCR on the same day. siSCR samples were normalised to 1.0. M = Mock, S = siSCR, E = siELF3. (c) Charts show (i) range of densitometry values comparing paired siSCR and siELF3 treated cells and also showing (ii) range of densitometry values across six days. One-way ANOVA with Tukey’s correction was used to compare the samples. Error bars are standard deviation of the mean.


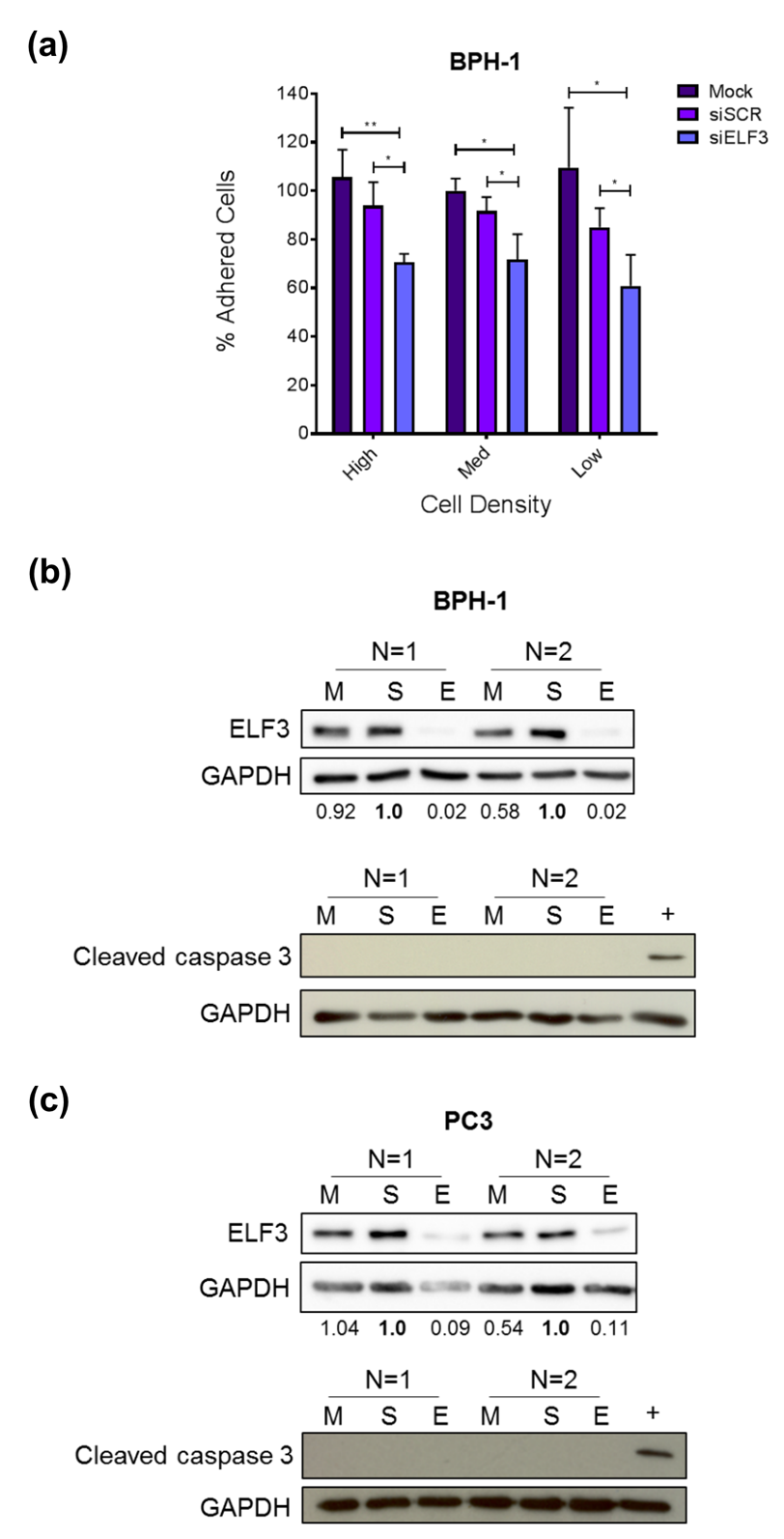


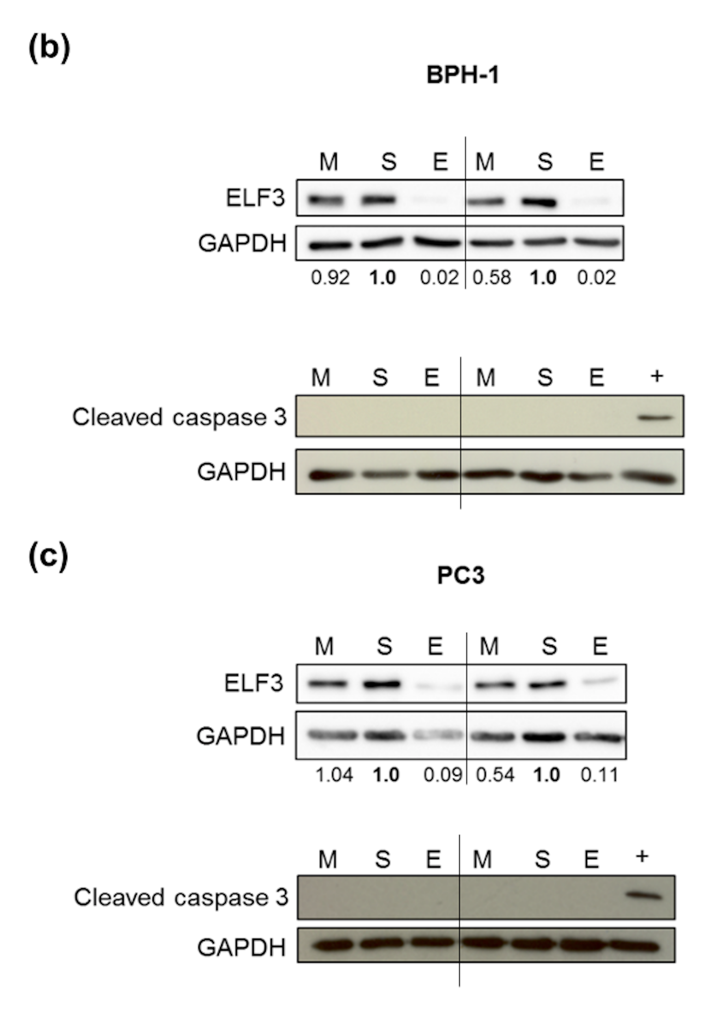


**Supplementary Figure 4 - ELF3 knockdown reduces cell adhesion but does not cause cell death via apoptosis.** (a) An adhesion assay was performed on BPH-1 cells 48h following knockdown (n=3). Cells were trypsinised and re-plated at three different densities for 4 hours (High = 300,000 cells, Med = 100,000 cells, Low = 40,000 cells). Floating cells were washed off and adherent cells were counted using a cell counter. Statistical significance was determined using a Student’s T-test (unpaired, two-tailed). Error bars are standard deviation of the mean. To assess cell death by apoptosis, lysates of (b) BPH-1 (n=2) and (c) PC3 (n=2) cells with ELF3 knockdown were probed for cleaved caspase 3. BPH-1 cells treated with 1μM Staurosporine for 24h were used as a positive control for apoptosis (+).


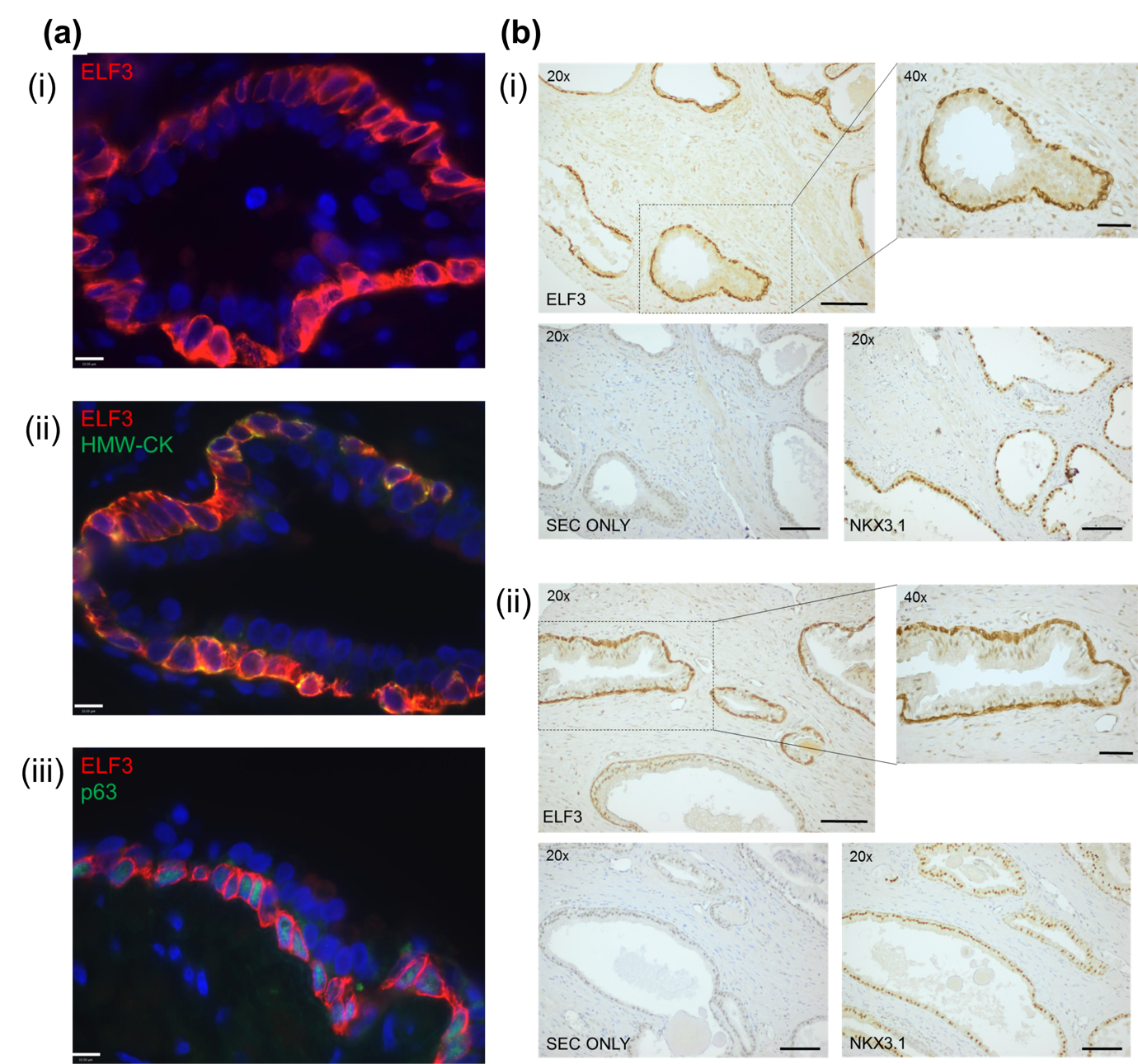


**Supplementary Figure 5. ELF3 Expression in BPH tissue.** (a) ELF3 expression was analysed in BPH tissue by immunohistochemistry (immunofluorescence). Formalin-fixed, paraffin-embedded BPH tissue was deparaffinised and rehydrated before undergoing heat-inducing antigen retrieval in sodium citrate buffer. IHC was carried out and sections were incubated with fluorescent Alexa Fluor secondary antibodies. (i) ELF3 alone (Ab97310), (ii) ELF3 co-stained with high molecular weight cytokeratin (cytoplasmic basal cell marker) and (iii) ELF3 co-stained with p63 (nuclear basal cell marker. Red = ELF3, Green = HMW-CK / p63, Blue = DAPI. 60x oil lens. Scale bar = 10μm. (b) ELF3 expression was analyed in BPH tissue microarrays (TMAs). 102 tissue sections from 40 patients were stained for ELF3 expression using Ab97310 using the Vector ImPRESS Excel Kit. Representative images of sections from two patients are shown. (i) Patient 9C exhibits more cytoplasmic staining whilst (ii) Patient 5A exhibits more nuclear staining. Sections were stained for Nkx3.1 as a nuclear luminal cell control. Sec only = tissues stained with secondary antibody only. 20x scale bar = 100μm, 40x scale bar = 50μm.


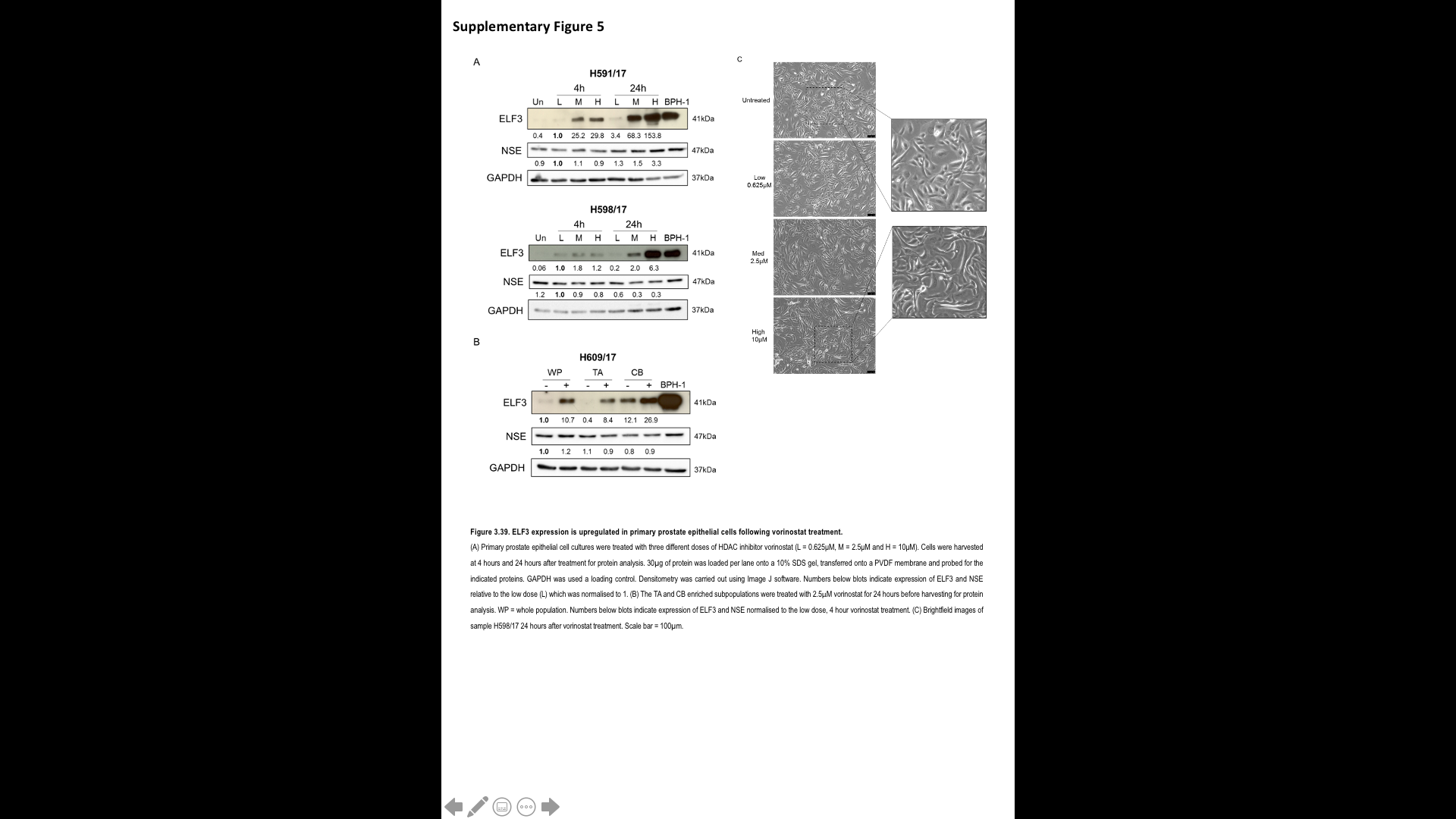


(c)

(b)

(a)

**Supplementary Figure 6: ELF3 expression is upregulated in primary prostate epithelial cells following vorinostat treatment.** (a) Primary prostate epithelial cell cultures were treated with three different doses of HDAC inhibitor vorinostat (L = 0.625μM, M = 2.5μM and H = 10μM). Cells were harvested at 4 hours and 24 hours after treatment for protein analysis. Numbers below blots indicate expression of ELF3 and NSE relative to the low dose (L) which was normalised to 1. (b) The TA and CB enriched subpopulations were treated with 2.5μM vorinostat for 24 hours before harvesting for protein analysis. WP = whole population. Numbers below blots indicate expression of ELF3 and NSE normalised to the low dose, 4 hour vorinostat treatment. (c) Brightfield images of sample H598/17 24 hours after treatment. Scale bar = 100μm.


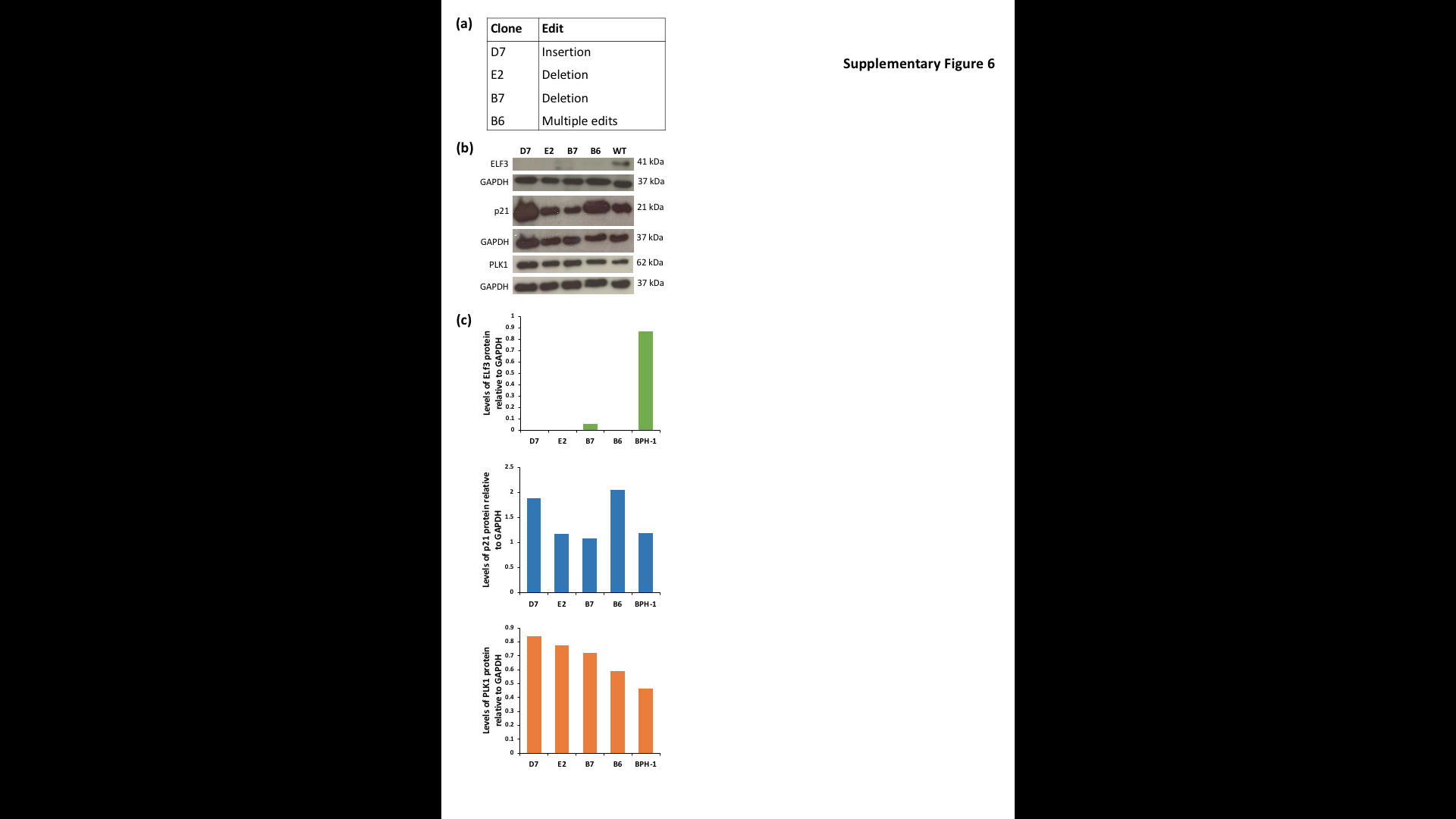


**Supplementary Figure 7. Impact of ELF3 CRISPR knockout on cell cycle markers.** ELF3 was knocked down in BPH-1 cells using a CRISPR lentivirus. (a) Four clones were examined for type of genetic alteration that occurred (b) Protein expression levels for ELF3, p21 and PLK1 were measured in each clone using Western Blot analysis. (c) Intensity of bands on Western blot was analysed to determine change in expression of ELF3, p21 and PLK1 in ELF3 clones.

**Supplementary Table 1. Antibodies used for protein detection by Western blot.**

**
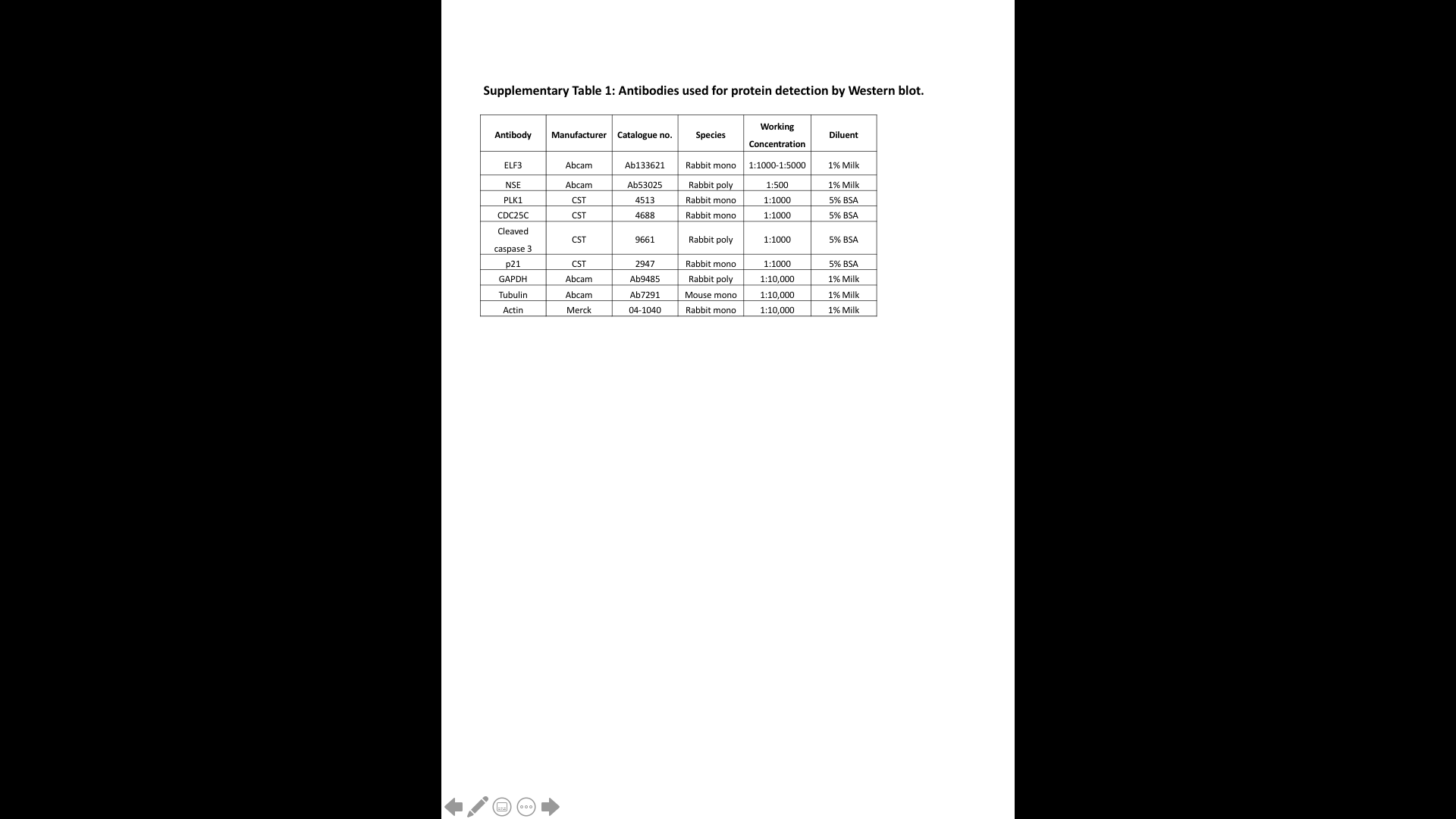
**

**Supplementary Table 2. Antibodies used for protein detection by Immunohistochemistry.**

**
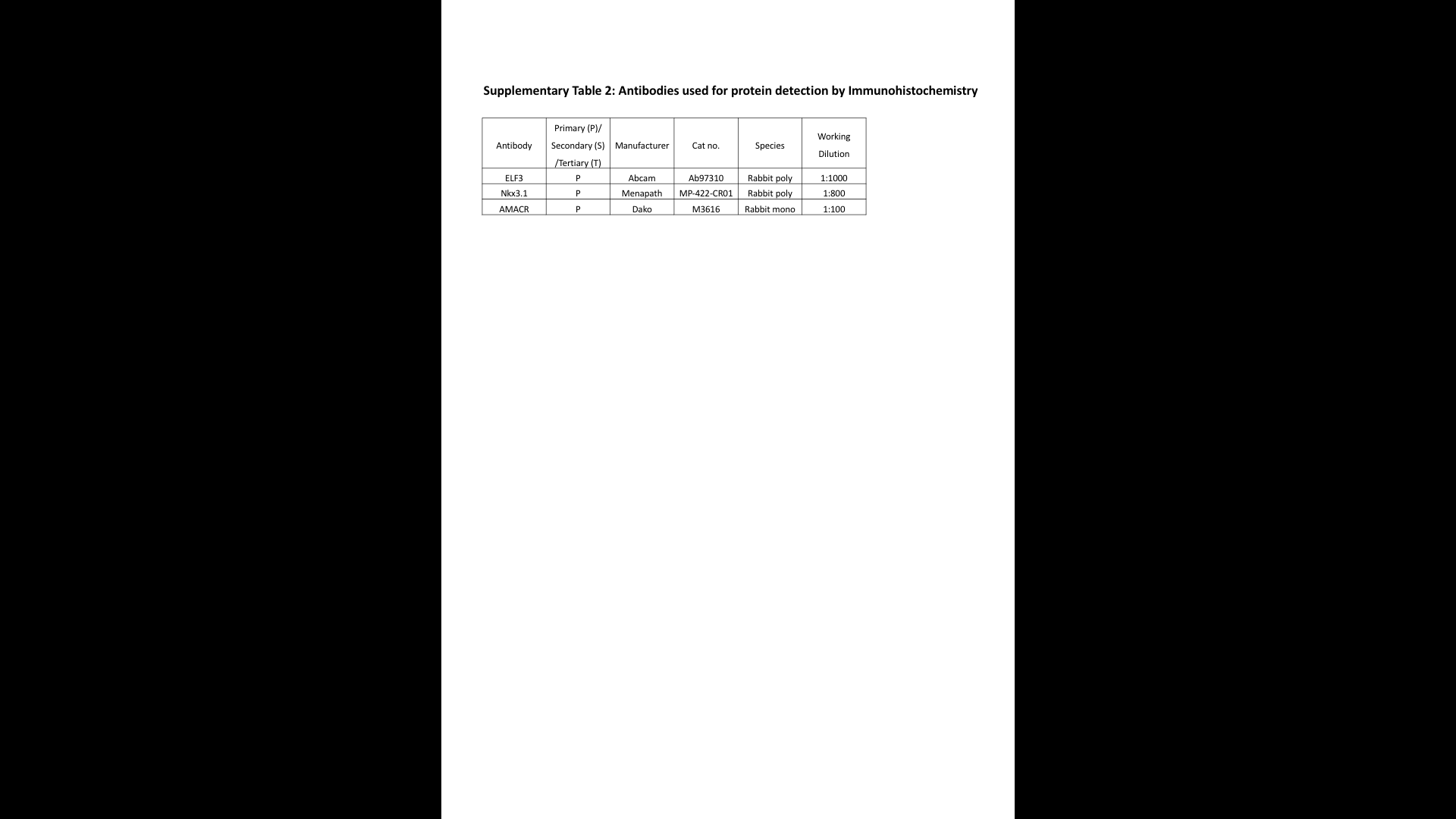
**

**Supplementary Table 3. Antibodies used for protein detection by Immunocytochemistry.**

**
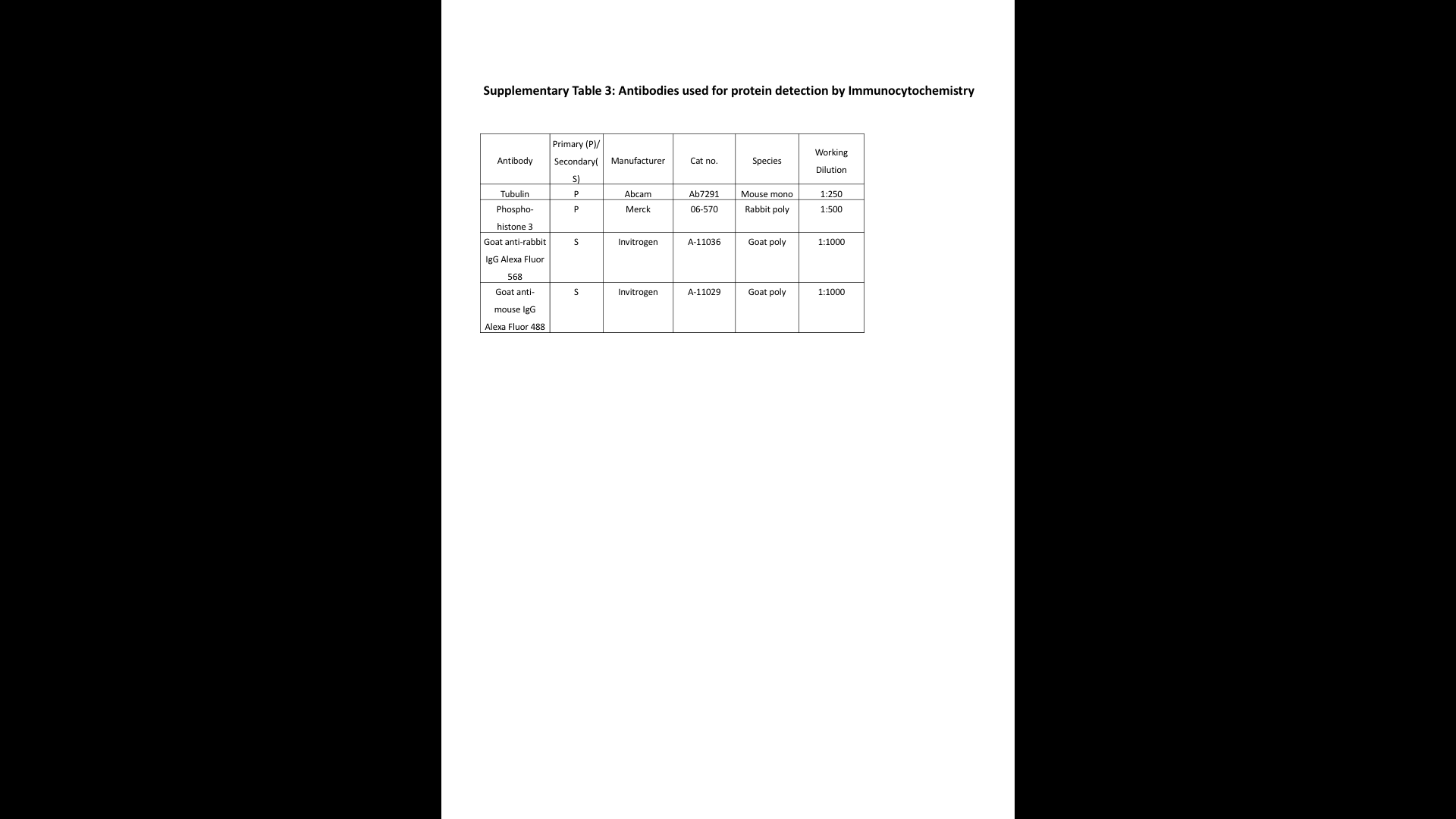
**

**Supplementary Table 4. Expression changes of cell cycle-related genes following ELF3 knockdown from gene expression microarray.** Highlighted boxes indicate genes with significance threshold of 2-fold increase or decrease and a p-value <0.05. NS = not significant. (ANOVA analysis with eBayes correction was used)

**
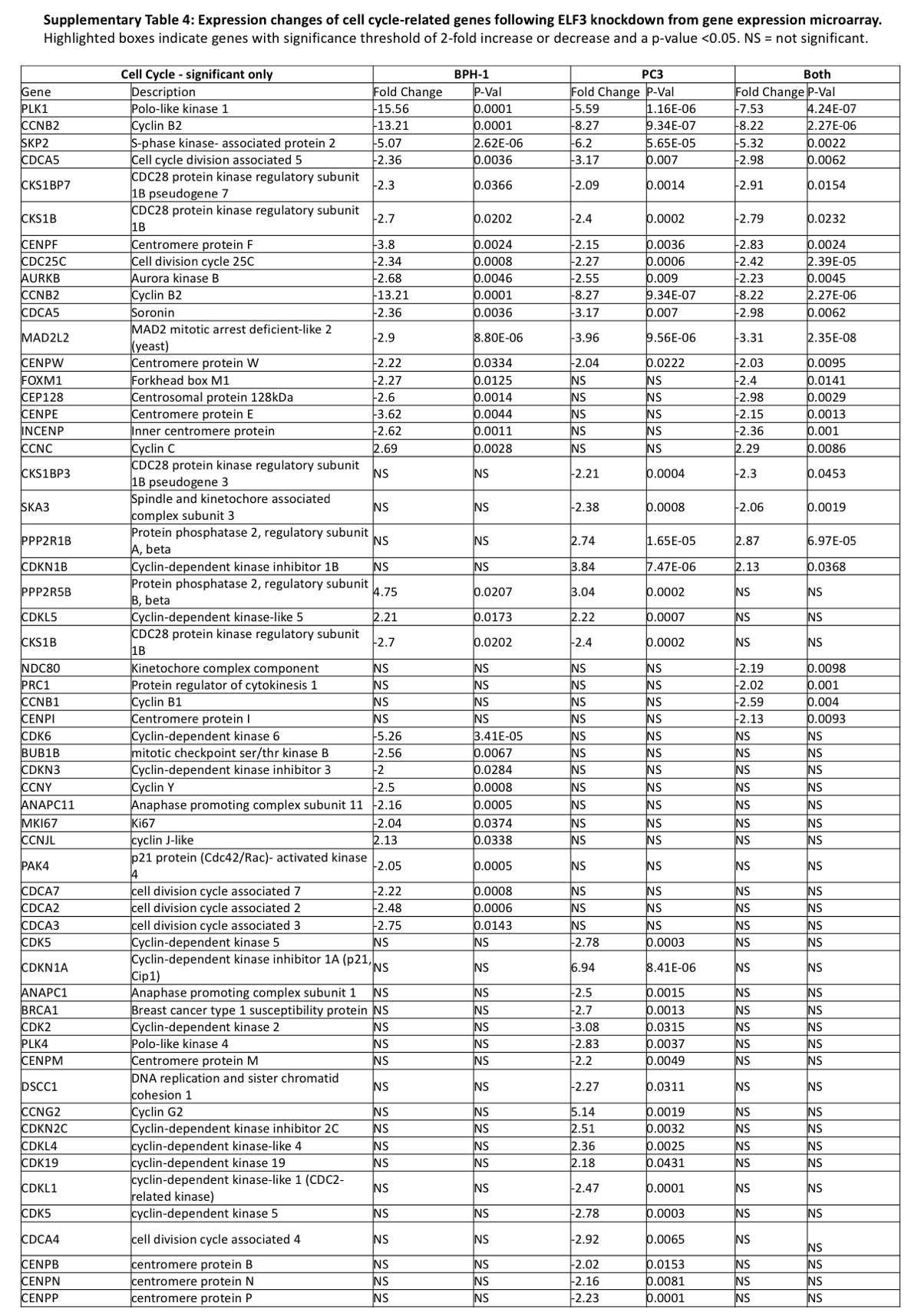
**

**Supplementary Table 5. List of studies used in Figure 8.**

**
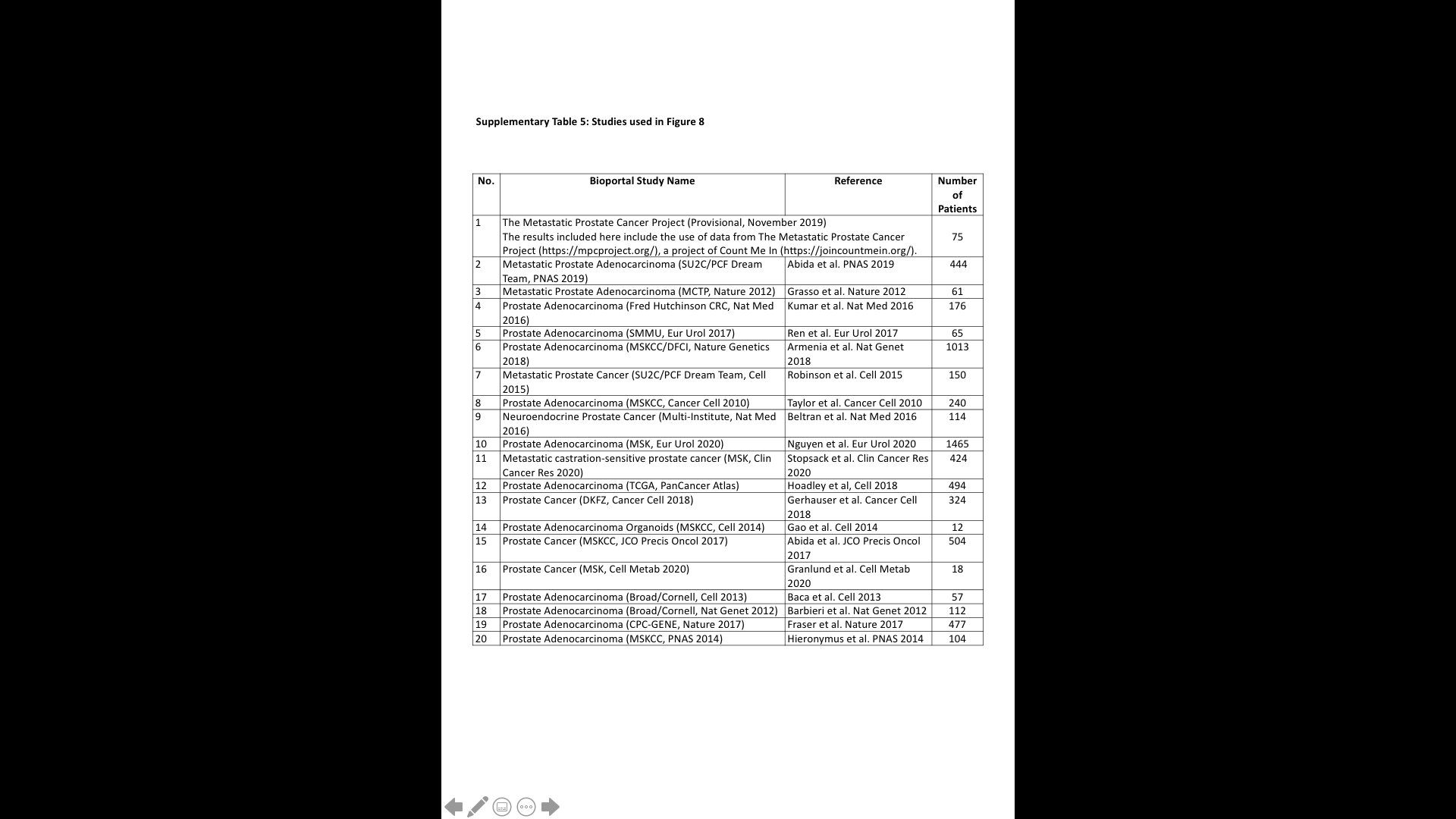
**
